# Supplementary material for: Classification and phylogeny for the annotation of novel eukaryotic GNAT acetyltransferases
Source: PLoS Comput Biol. 2020 Dec 23;16(12):e1007988. doi: 10.1371/journal.pcbi.1007988 (PMC7790372; doi:10.1371/journal.pcbi.1007988)
Supplement: S1 Text — (PDF) [file pcbi.1007988.s001.pdf]

General information about network construction and network topology

Right alignment score threshold ensures cluster isofunctionality

We used EFI-EST [1] to generate all SSNs, which allows use of the alignment score to control the network topology. Figure A shows percentage identity as function of alignment score, which eventually determines which nodes will be connected by an edge. Goals in constructing an SSN is can differ, and depending on that, the alignment score threshold will differ. In case an SSN is used estimate a number of enzymes with different specificities within the superfamily, a threshold should be higher. Conversely, if an SSN should estimate relationships between enzymes of different specificities (directions of sequence change through evolution), a lower threshold should be used. In addition to the desired goal of SSN, sequence divergence within the used dataset will dictate threshold selection. We opted for lower thresholds in our case, since acetyltransferases are extremely dissimilar in terms of sequence (**Fig A**).

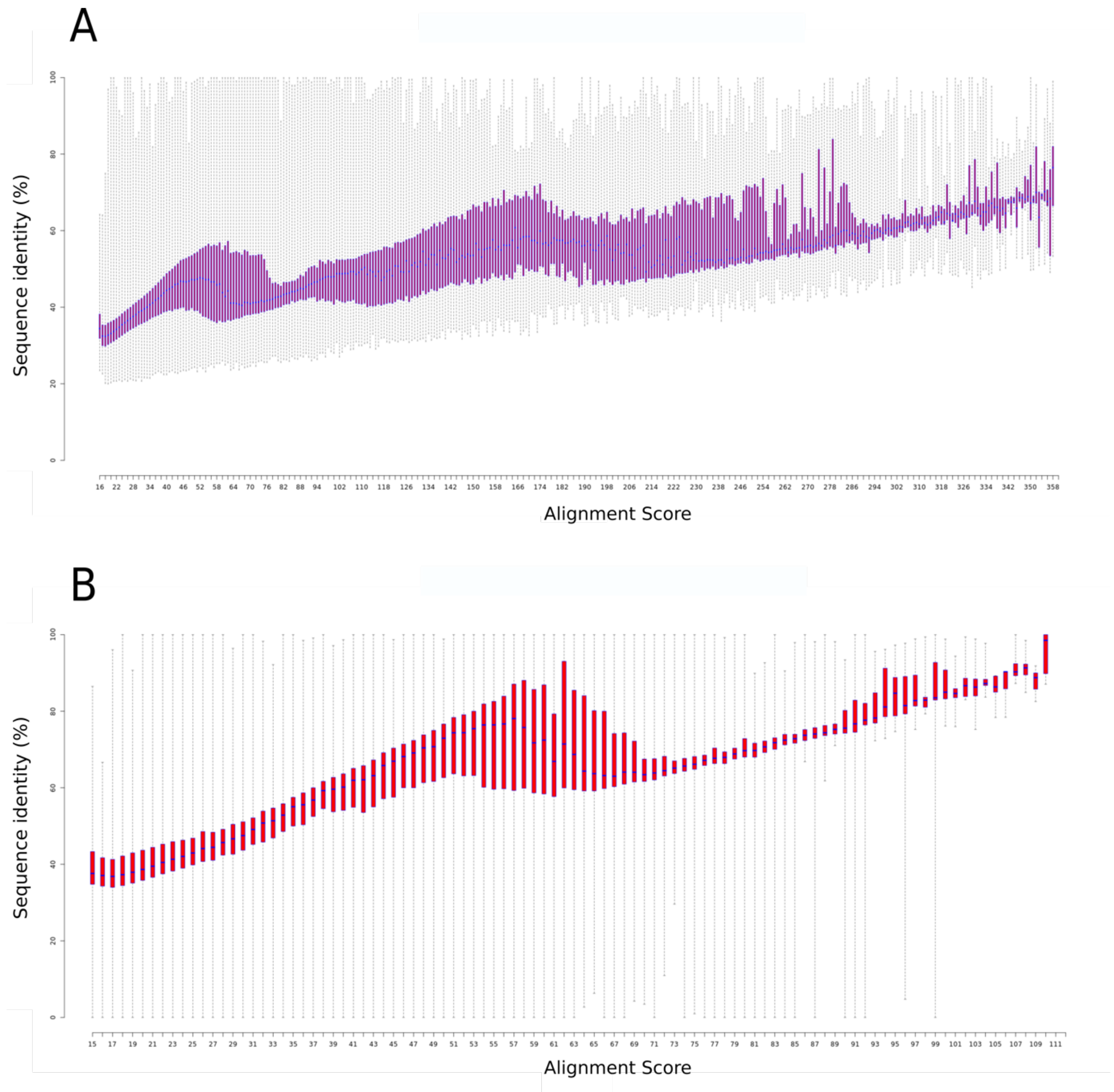

Fig A. Alignment score of 30 in the full-sequence network (**A**) and 21 in the GNAT domain SSN (**B**) ensures average percentage identity of ~40%. Using alignment scores of 30 and 21 for the full-sequence network and the GNAT fold network respectively also ensures similar characteristics in both of the networks. This allows comparing the two networks.

Diversity of the acetyltransferase superfamily

The SSNs obtained from both datasets (the full-sequence and only GNAT domain sequence) show large sequence divergence in the acetyltransferase superfamily (**Fig B**).

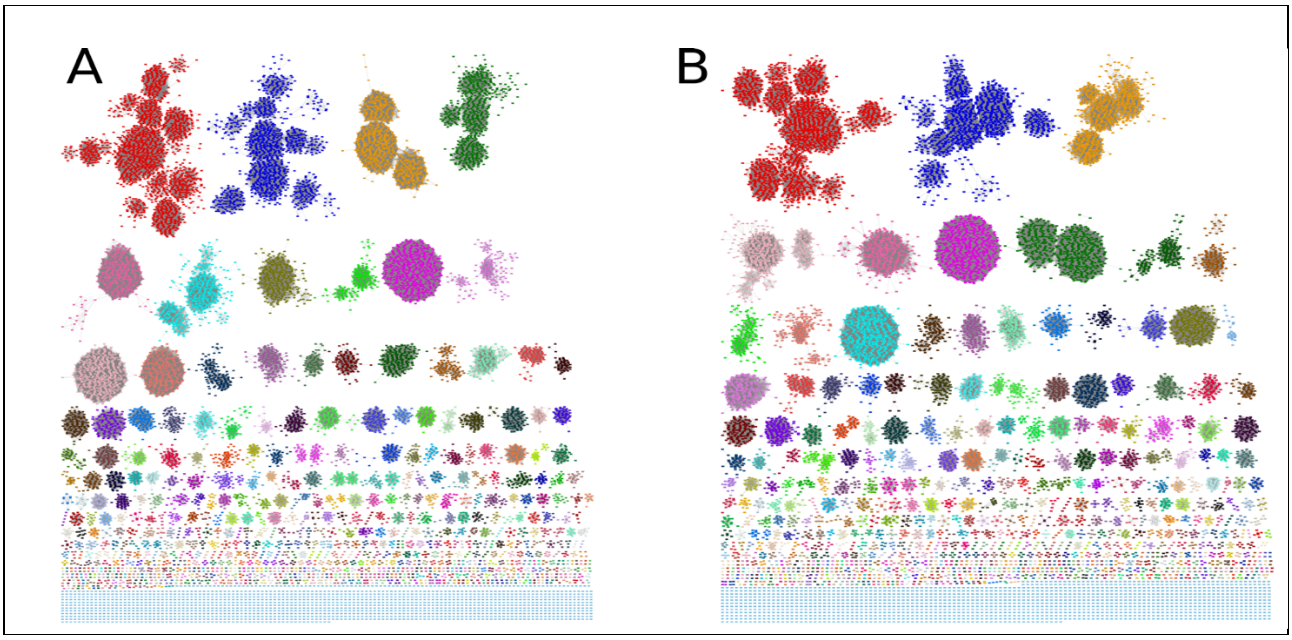

Fig B. Sequence similarity networks built using full-sequence (A) and GNAT domain (B) datasets. Each network is built from a large number of highly disconnected clusters. There are several connected components in each of the networks, which could represent distinct enzyme families within the superfamily, but since an SSN is a result of only pairwise alignments it is not possible to draw such a conclusion with higher degree of confidence.

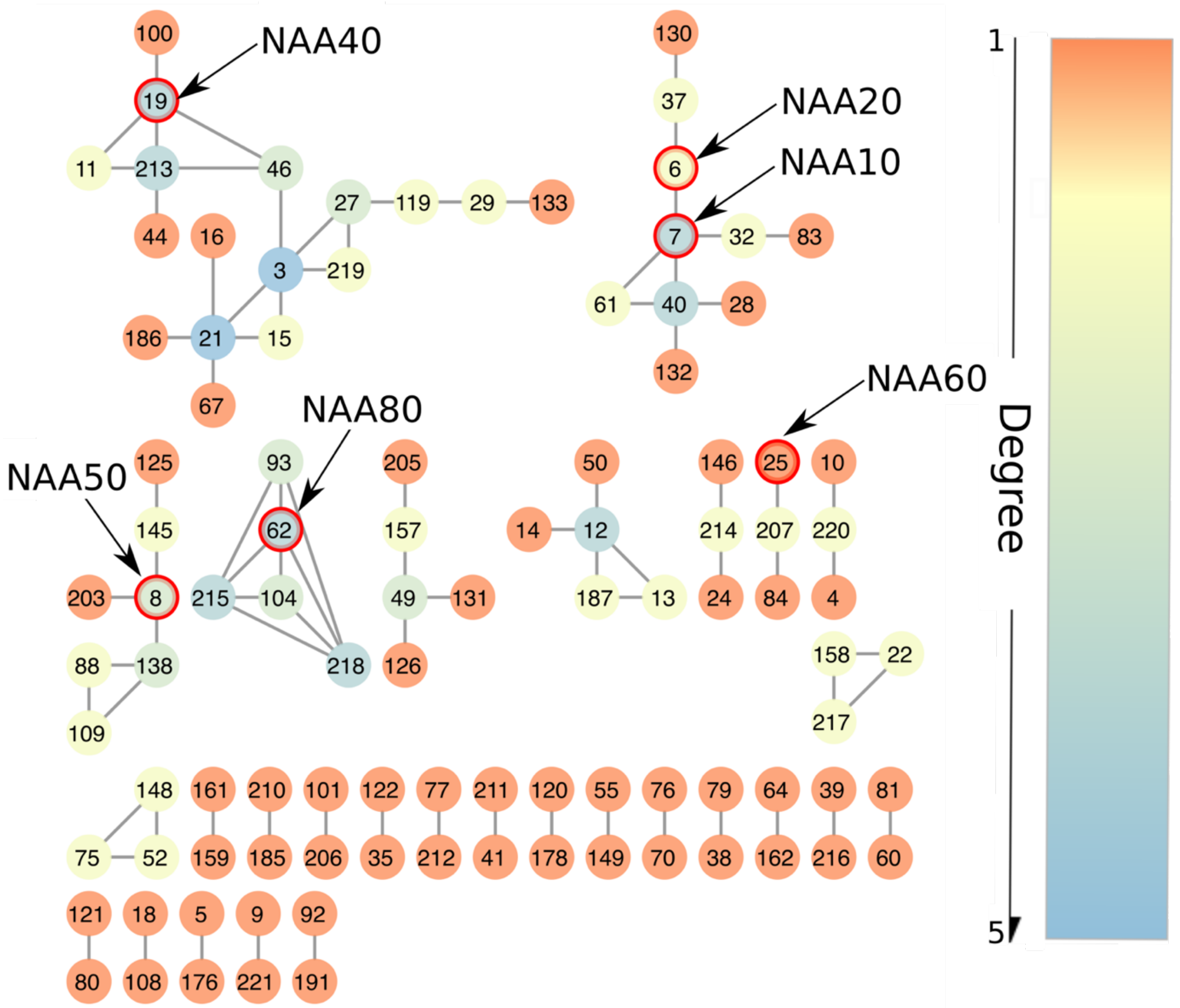

Fig C. Pivot network of GNAT domain portion of sequences. Interactions between clusters in GNAT domain network remain the same as they are in the full-sequence network. IMPORTANT: Cluster numbers in this network differ from those in the full-sequence SSN because different numbers were assigned to clusters by ClusterONE after network density decomposition was ran for each of the two networks.

References:

1. Gerlt JA, Bouvier JT, Davidson DB, Imker HJ, Sadkhin B, Slater DR, et al. Enzyme Function Initiative-Enzyme Similarity Tool (EFI-EST): A web tool for generating protein sequence similarity networks. Biochim Biophys Acta - Proteins Proteomics. 2015 Aug;1854(8):1019–37.
