# Supplementary material for: Classification and phylogeny for the annotation of novel eukaryotic GNAT acetyltransferases
Source: PLoS Comput Biol. 2020 Dec 23;16(12):e1007988. doi: 10.1371/journal.pcbi.1007988 (PMC7790372; doi:10.1371/journal.pcbi.1007988)
Supplement: S2 Text — (PDF) [file pcbi.1007988.s002.pdf]

There are at least 5 groups of N-terminal acetyltransferases, according to SSN clustering

Group 1 – Group 1a: NAA10, NAA20 and Group 1b: NAA30

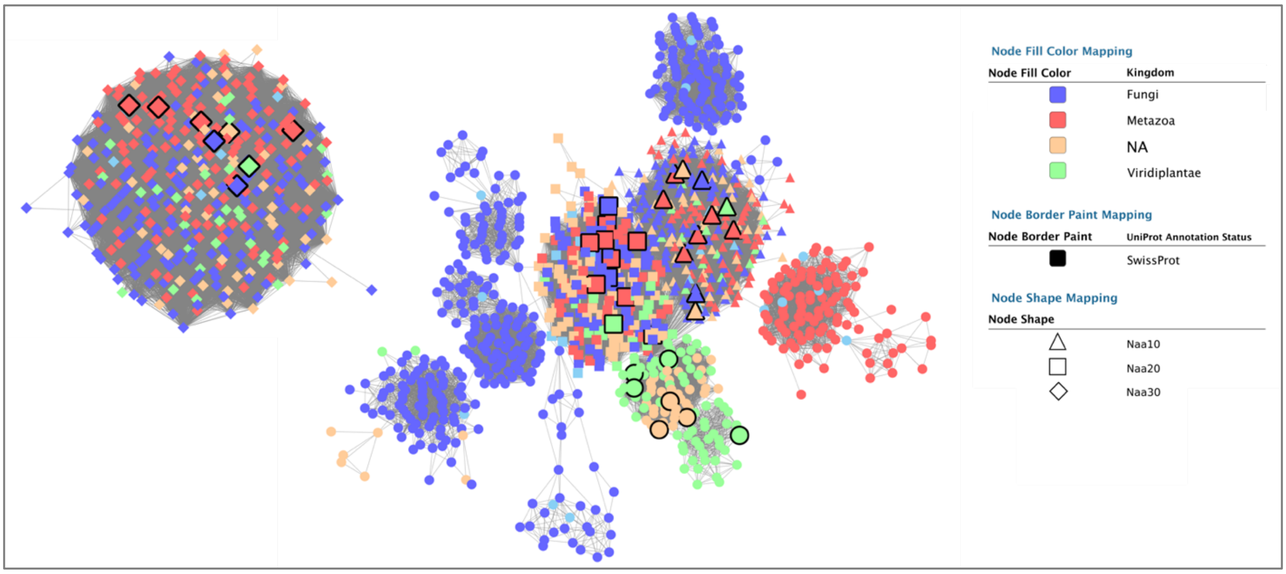

Fig A. Clustering around NAA10 and NAA20 is not based on taxonomy. NAA30 forms a single cluster, not connected to the rest of the network. This figure clearly shows that sequences of the same specificity cluster together, regardless of their taxonomy. Larger nodes with black outline are sequences from SwissProt – reviewed sequences. All other nodes are sequences from TrEMBL – uncharacterized sequences or automatically annotated.

Group 2 – NAA50 and NAA60

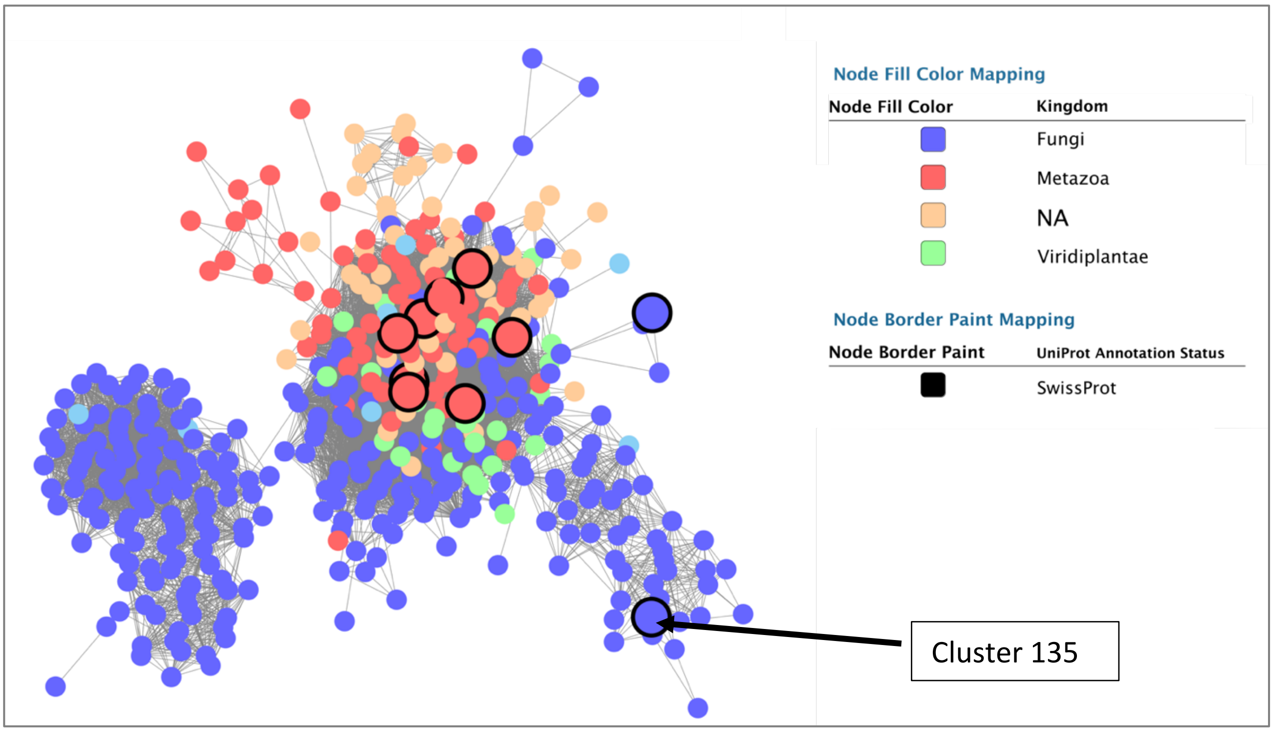

Fig B. Stringent network (E-value 10^{-15}, alignment score 30). Clustering in Group 2 around NAA50 is not based on taxonomical differences. Larger nodes with black outline represent sequences from SwissProt. One reviewed sequence form cluster 135, even though annotated as NAA50, has specificity different than human NAA50 (Van Damme et al. 2015).

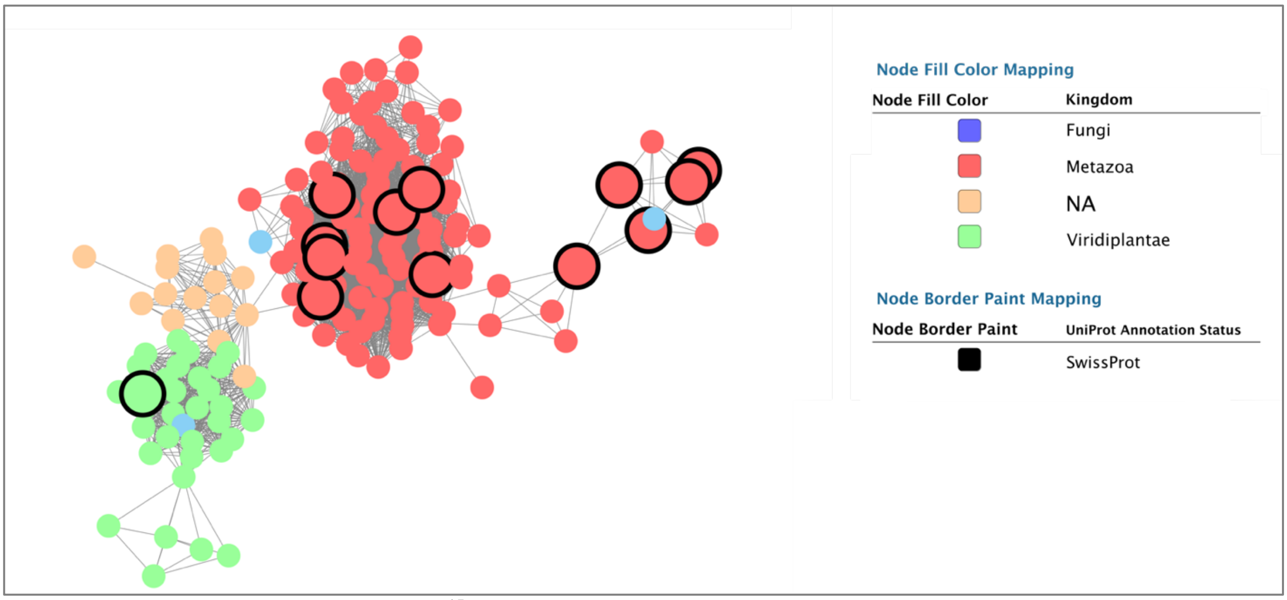

Fig C. Stringent network (E-value 10^{-15}, alignment score 30). Clustering in Group 2 around NAA60 is based on taxonomical differences. Larger nodes with black outline represent sequences from SwissProt. NAA60 has been secondarily lost in fungi. All reviewed sequences are annotated as NAA60.

Group 3 – NAA40

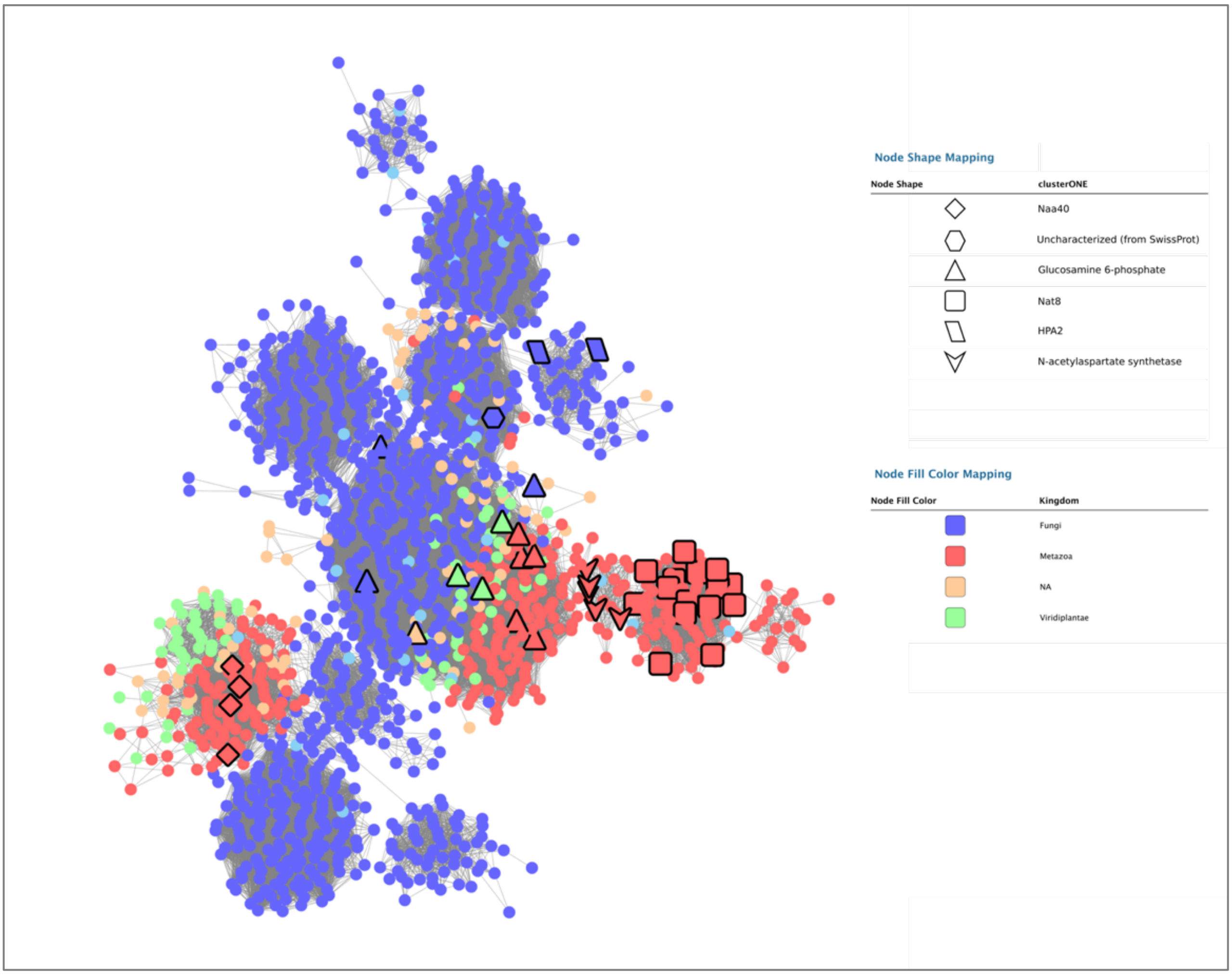

Fig D. Stringent network (E-value 10^{-15}, alignment score 30). NAA40 clusters into the same connected component with several other non-NAT acetyltransferases. This connected component is dominated mainly by proteins from fungi,

but clustering doesn't seem to be affected by taxonomy as we can see in several regions where sequences from different kingdoms cluster together.

Group 4 – NAA80

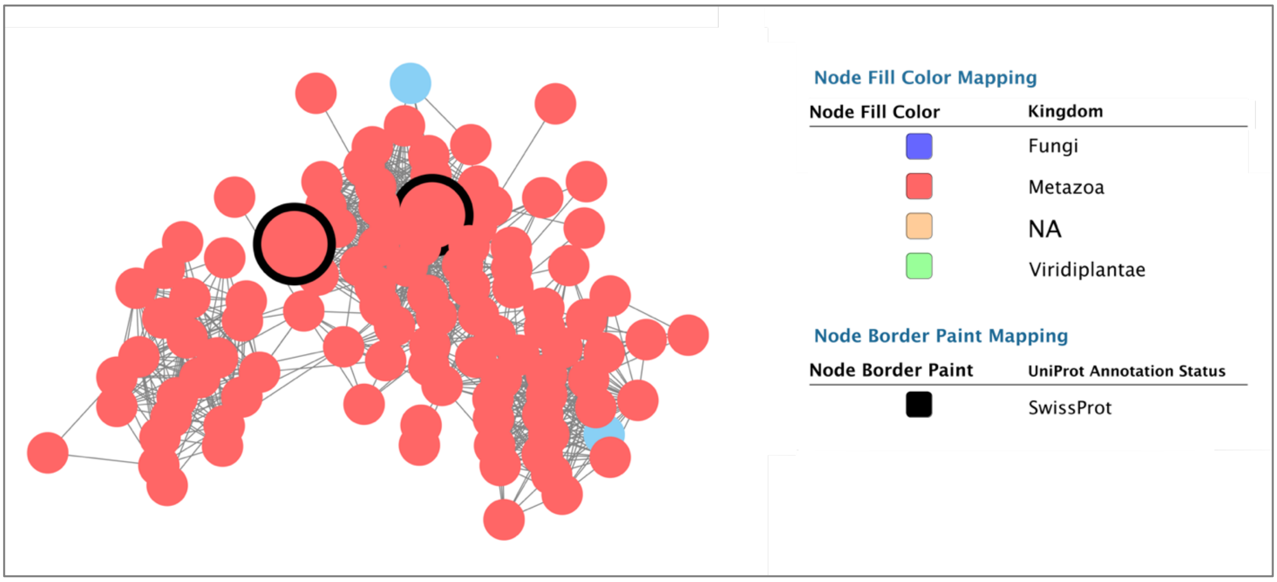

Fig E. Stringent network (E-value 10^{-15}, alignment score 30). NAA80 is present only in animal kingdom.
