# Supplementary material for: Classification and phylogeny for the annotation of novel eukaryotic GNAT acetyltransferases
Source: PLoS Comput Biol. 2020 Dec 23;16(12):e1007988. doi: 10.1371/journal.pcbi.1007988 (PMC7790372; doi:10.1371/journal.pcbi.1007988)
Supplement: S3 Text — (PDF) [file pcbi.1007988.s003.pdf]

Multiple sequence alignments in the N-terminal acetyltransferase family

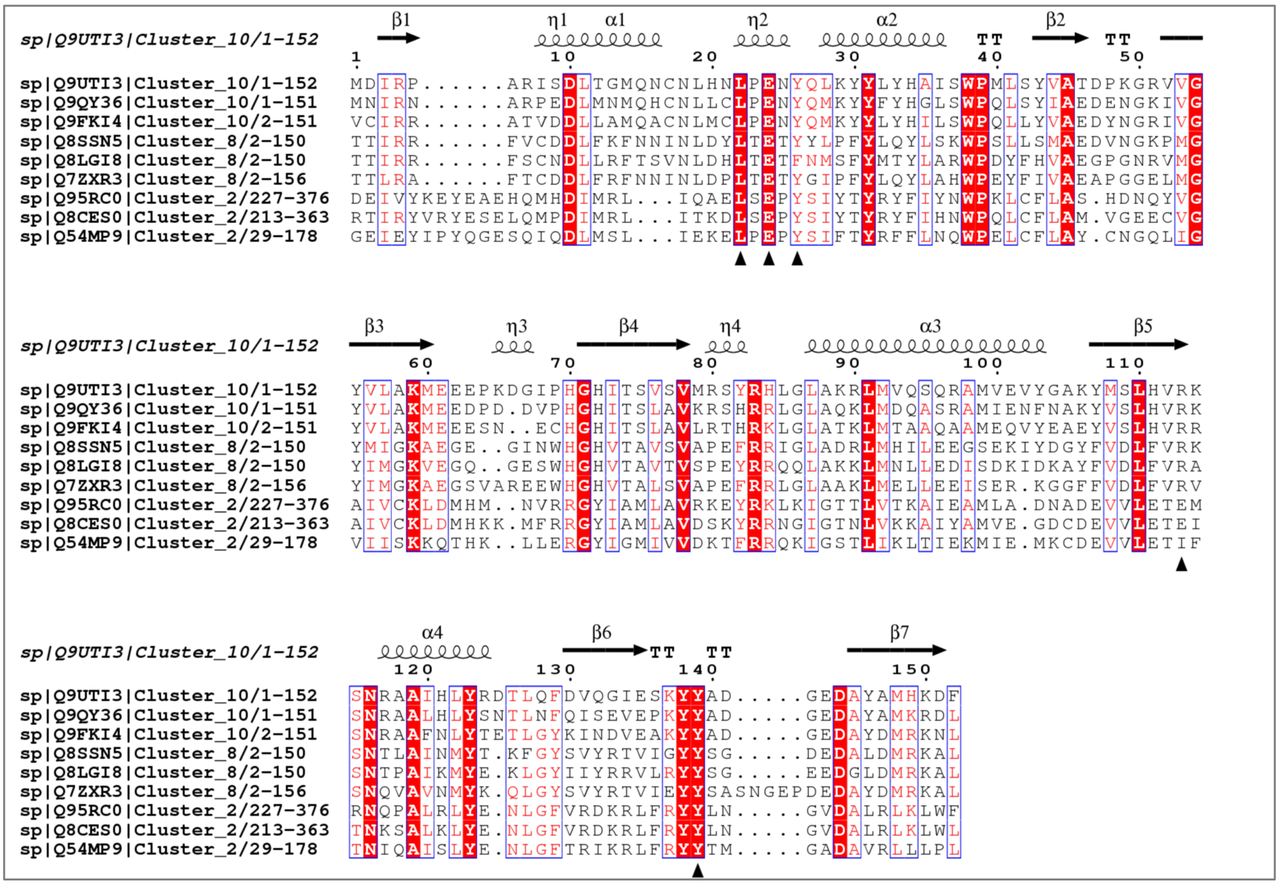

Fig A. Multiple sequence alignment of NAA10 (cluster 10), NAA0 (cluster 8) and NAA30 (cluster 2). Group 1 of NATs with its two subgroups (Group 1a: NAA10 (Cluster10) and NAA20 (Cluster 8), Group 1b: NAA30 (Cluster 2). Black triangles mark key residues in Group I of NATs. Key residues are conserved in all three NATs, except R113 of NAA10, which is conserved only in NAA20, but not in NAA30. Nevertheless, a negatively charged residue in this position in NAA30 is involved in substrate recognition.

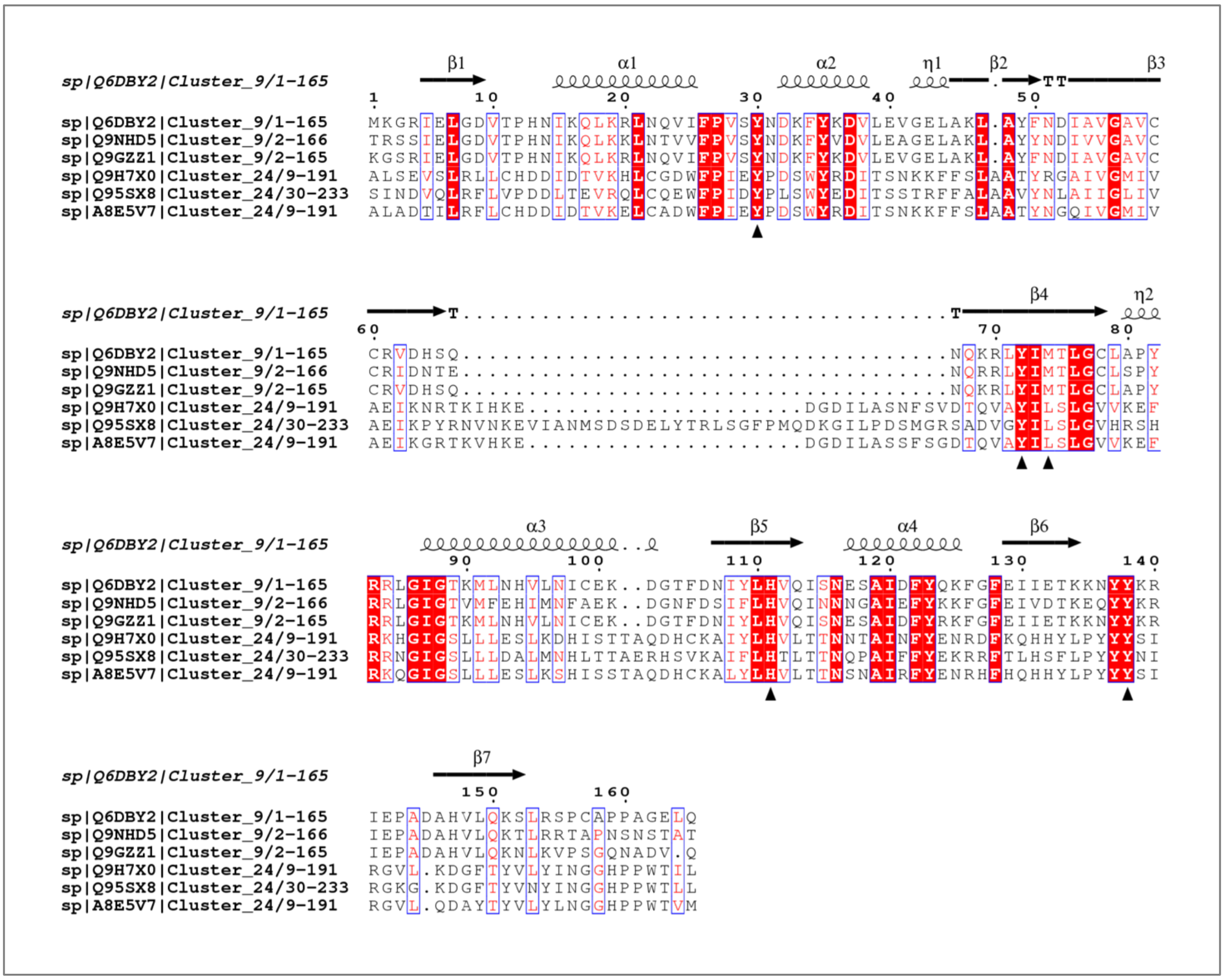

Fig B. Multiple sequence alignment of NAA50 (cluster 9) and NAA60 (cluster 24). Group 2 of NATs. NAA50 and NAA60 are highly conserved and have even been shown to acetylate similar substrates. Black triangles mark residues important for substrate binding and catalysis. All of these residues are highly conserved between NAA50 and NAA60.

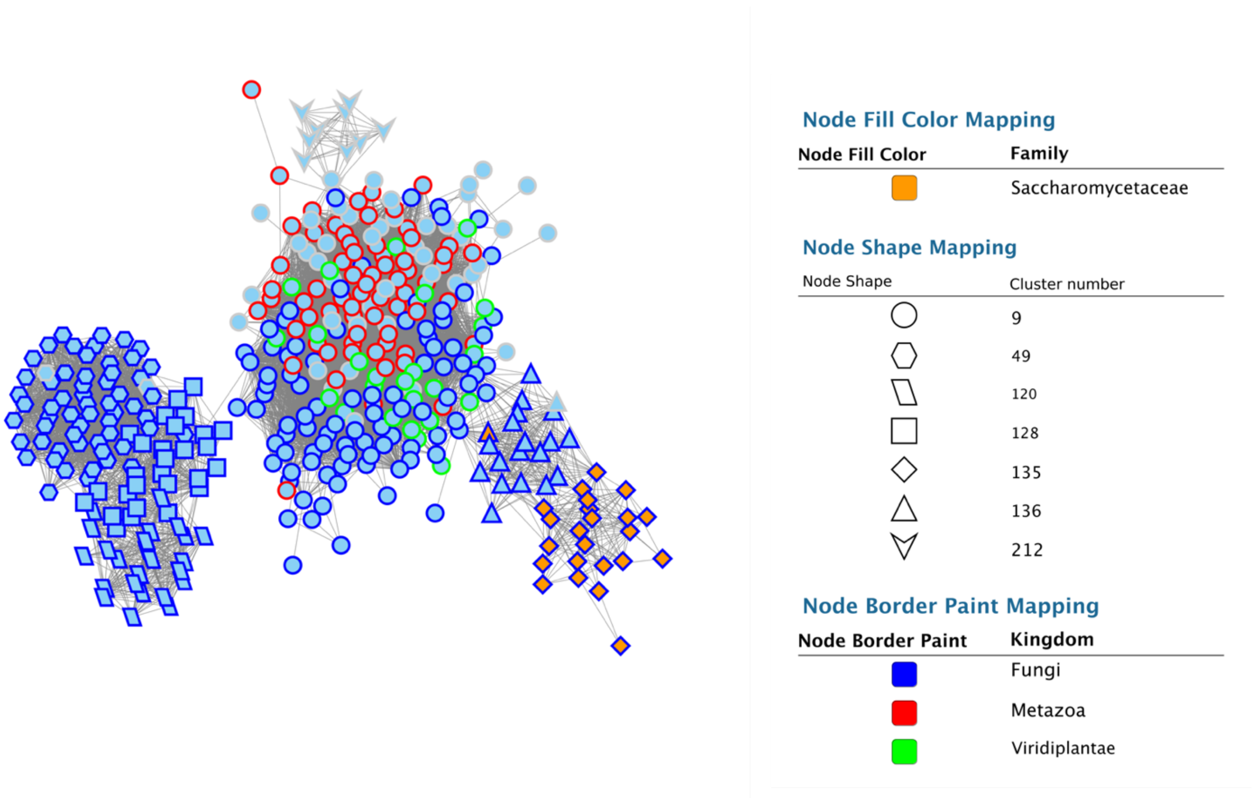

Fig C. Cluster 135 consists of inactive yeast NAA50 which is a member of the Group 2 NATs. NAA50 from fungi, plants and animals form a cluster together – cluster 9. All of these sequences share the same key sequence motifs. An inactive yeast NAA50 forms cluster 135. This protein doesn't have the conserved binding sequence motifs and the difference is reflected in clustering. Interestingly, the absence of key sequence motifs is a characteristic of one fungal family – Saccharomycetaceae.

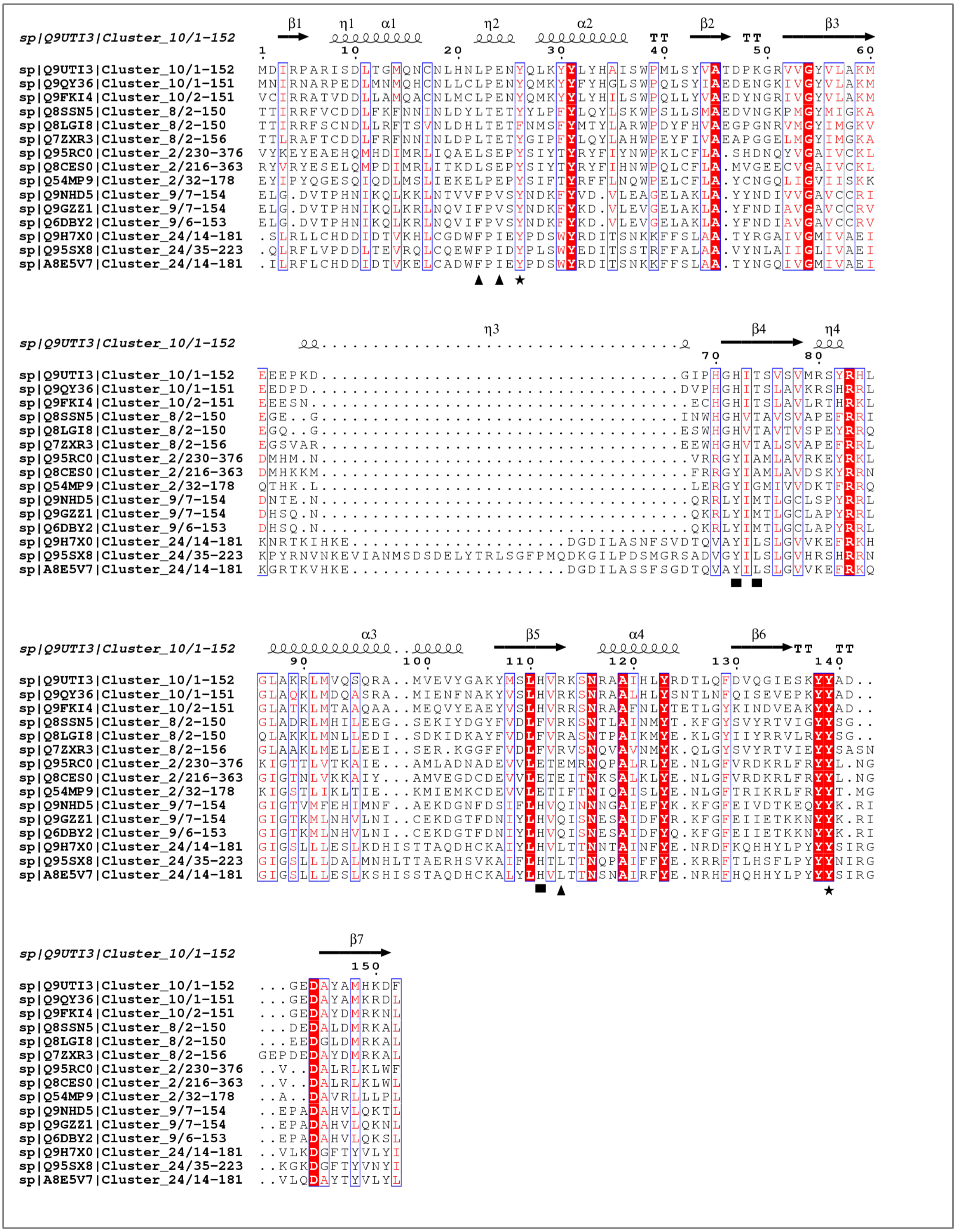

Fig D. Multiple sequence alignment of NAA10, NAA20, NAA30, NAA50 and NAA60. Groups 1 and 2 show significant conservation, even though they acetylate different substrates. Black triangles mark important residues in Groups 1a and 1b (NAA10, NAA20 and NAA30). Black squares mark residues important for substrate binding and catalysis in Group 2 (NAA50 and NAA60). Black stars mark residues involved in catalysis and substrate binding in both groups.
