# Supplementary material for: Classification and phylogeny for the annotation of novel eukaryotic GNAT acetyltransferases
Source: PLoS Comput Biol. 2020 Dec 23;16(12):e1007988. doi: 10.1371/journal.pcbi.1007988 (PMC7790372; doi:10.1371/journal.pcbi.1007988)
Supplement: S4 Text — (PDF) [file pcbi.1007988.s004.pdf]

Small-world network

It is not possible to learn much about relationships between different, less similar acetyltransferases from the stringent network we used for finding isofunctional clusters. In order to learn more about the relationship between known NATs and the rest of the superfamily we had to find a suitable less stringent network. A suitable less stringent network is the one with a large group of connected clusters that exhibits small-world characteristics (a random network or a lattice network do not offer any useful information since they have too many or too few edges between nodes). Networks with small-world characteristics have been shown to be able to describe real-world systems [1].

We calculated smallworldness of 21 different networks (**Table A**) and found that the most suitable one for inferring relationships between acetyltransferases is the network constructed with the following parameters: E-value = 10^{-10}, alignment score = 15. This network has 14 connected components with the largest one containing 144 clusters (**Fig A**). Smallworldness for the largest connected component of this network was calculated using two different metrics implemented through networkx, a python library for studying graphs and networks. The first metric is omega [2] and the second one is sigma [3]. Omega for the largest connected component for the network with E-value of 10^{-10} and alignment score of 15 was calculated to be -0,37, while sigma was calculated to be 3,4. Both of these results indicate that the network has small-world characteristics. List of omega and sigma results for every measured network in (**Table A**).

All of the known NATs, except the plastid NAT identified in *Arabidopsis thaliana* [4], are present in the largest connected component of this network (**Fig A**), which makes it possible to explore their relationships among each other and with the rest of the superfamily. We included representative sequences of 142/144 clusters found in the largest connected component into a dataset for creating a phylogenetic tree of the acetyltransferase superfamily.

Table A. Searching for the largest possible connected component that has smallworld characteristics in SSNs constructed with various parameters. Sigma and omega, calculated using NetworkX Python module, are smallworldness indicators. Omega close to 0 means a graph is classified as smallworld. If sigma is larger than 1, a graph is classified as smallworld.

network name	number of components	size of largest component	sigma	omega
Eukaryota_dataset_with_all_human_ev5als40	23	11	1.413	-0.254
Eukaryota_dataset_with_all_human_ev5als15	15	148	3.225	-0.398
Eukaryota_dataset_with_all_human_ev5als35	29	14	1.582	-0.926
Eukaryota_dataset_with_all_human_ev5als25	21	64	7.216	-0.281
Eukaryota_dataset_with_all_human_ev5als30	27	22	2.411	-0.557
Eukaryota_dataset_with_all_human_ev5als20	20	100	4.356	-0.294
Eukaryota_dataset_with_all_human_ev10als25	21	64	5.955	-0.307
Eukaryota_dataset_with_all_human_ev10als20	20	100	4.486	-0.321
Eukaryota_dataset_with_all_human_ev10als30	27	22	2.746	-0.596
Eukaryota_dataset_with_all_human_ev10als35	29	14	1.742	-1.457
Eukaryota_dataset_with_all_human_ev10als40	23	11	1.411	-0.352
Eukaryota_dataset_with_all_human_ev10als15	14	144	3.445	-0.375
Eukaryota_dataset_with_all_human_ev15als16	20	110	2.796	-0.508
Eukaryota_dataset_with_all_human_ev15als25	21	64	6.707	-0.273
Eukaryota_dataset_with_all_human_ev15als30	27	22	2.682	-0.625
Eukaryota_dataset_with_all_human_ev15als35	29	14	1.772	-1.188
Eukaryota_dataset_with_all_human_ev15als40	23	11	1.352	-0.367
Eukaryota_dataset_with_all_human_ev20als20	21	72	4.631	-0.375
Eukaryota_dataset_with_all_human_ev20als30	27	22	2.817	-0.545
Eukaryota_dataset_with_all_human_ev20als40	23	11	1.321	-0.203
Eukaryota_dataset_with_all_human_ev20als35	29	14	1.849	-1.065

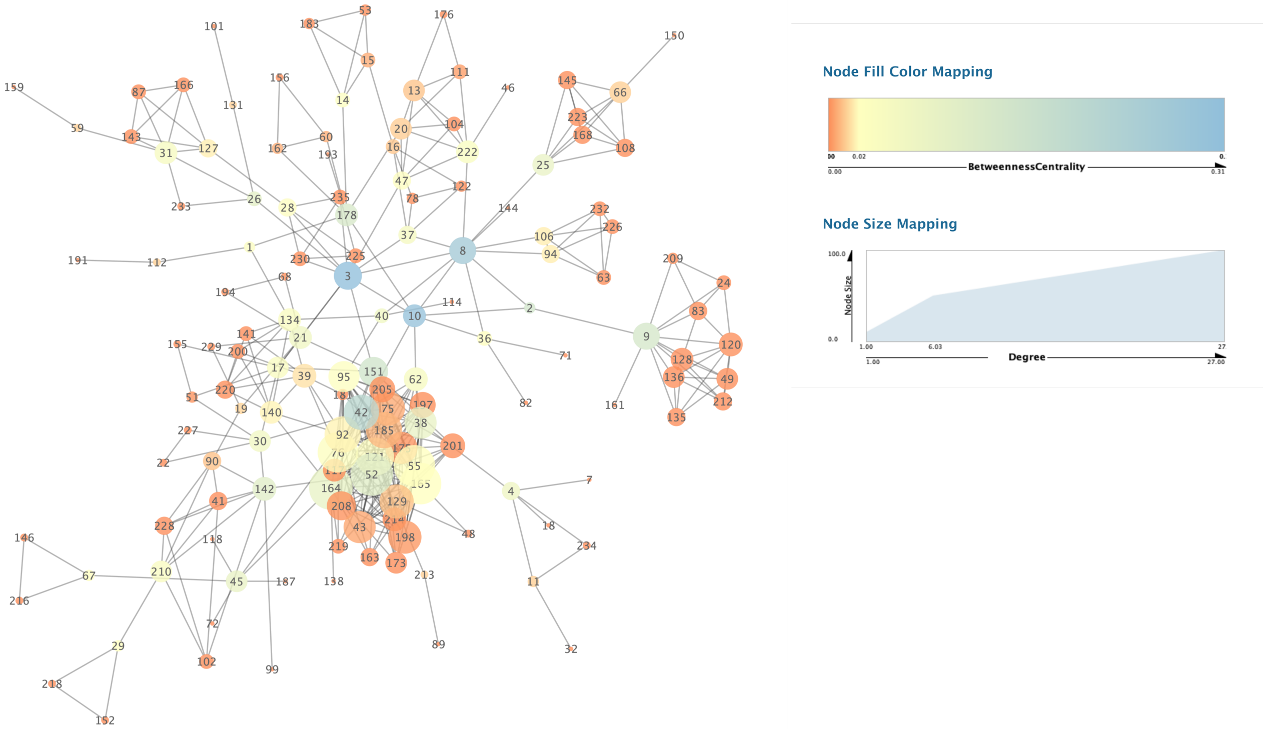

Fig A. Largest connected component for the network with E-value of 10^{-10} and alignment score of 15 exhibits small-world characteristics. It offers insight into the relationship of known NATs to one another and to the rest of the family.

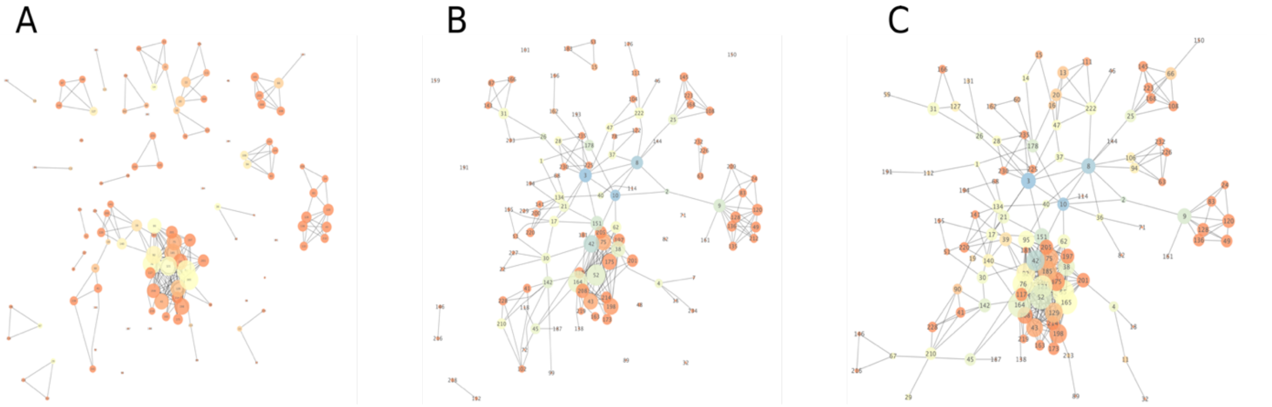

Fig B. Largest connected component of the SSN created with the E-value of 10^{-10} and the alignment score of 15 exhibits smallworld characteristics. If we remove the top 20% of nodes with the highest betweenness centrality (the hubs) the network becomes sparse (**A**). Since hubs connect distant parts of the network, removing them results in disrupting the interconnection of the network. On the other hand, removing nodes with lower betweenness centrality than the hubs (**B**) or nodes with betweenness centrality of 0 (**C**) the information in the network remains almost intact. This illustrates the importance of the hubs in our SSN.

References:

1. Valavanis I, Spyrou G, Nikita K. A similarity network approach for the analysis and comparison of protein sequence/structure sets. J Biomed Inform. 2010;43(2):257–67.
2. Telesford QK, Joyce KE, Hayasaka S, Burdette JH, Laurienti PJ. The Ubiquity of Small-World Networks. Brain Connect. 2011 Nov 14;1(5):367–75.
3. Humphries MD, Gurney K. Network 'Small-World-Ness': A Quantitative Method for Determining Canonical Network Equivalence. Sporns O, editor. PLoS One. 2008 Apr 30;3(4):e0002051.
4. Dinh T V., Bienvenut W V., Linster E, Feldman-Salit A, Jung VA, Meinnel T, et al. Molecular identification and functional characterization of the first Nα-acetyltransferase in plastids by global acetylome profiling. Proteomics. 2015;15(14):2426–35.
