# Supplementary material for: Classification and phylogeny for the annotation of novel eukaryotic GNAT acetyltransferases
Source: PLoS Comput Biol. 2020 Dec 23;16(12):e1007988. doi: 10.1371/journal.pcbi.1007988 (PMC7790372; doi:10.1371/journal.pcbi.1007988)
Supplement: S5 Text — (PDF) [file pcbi.1007988.s005.pdf]

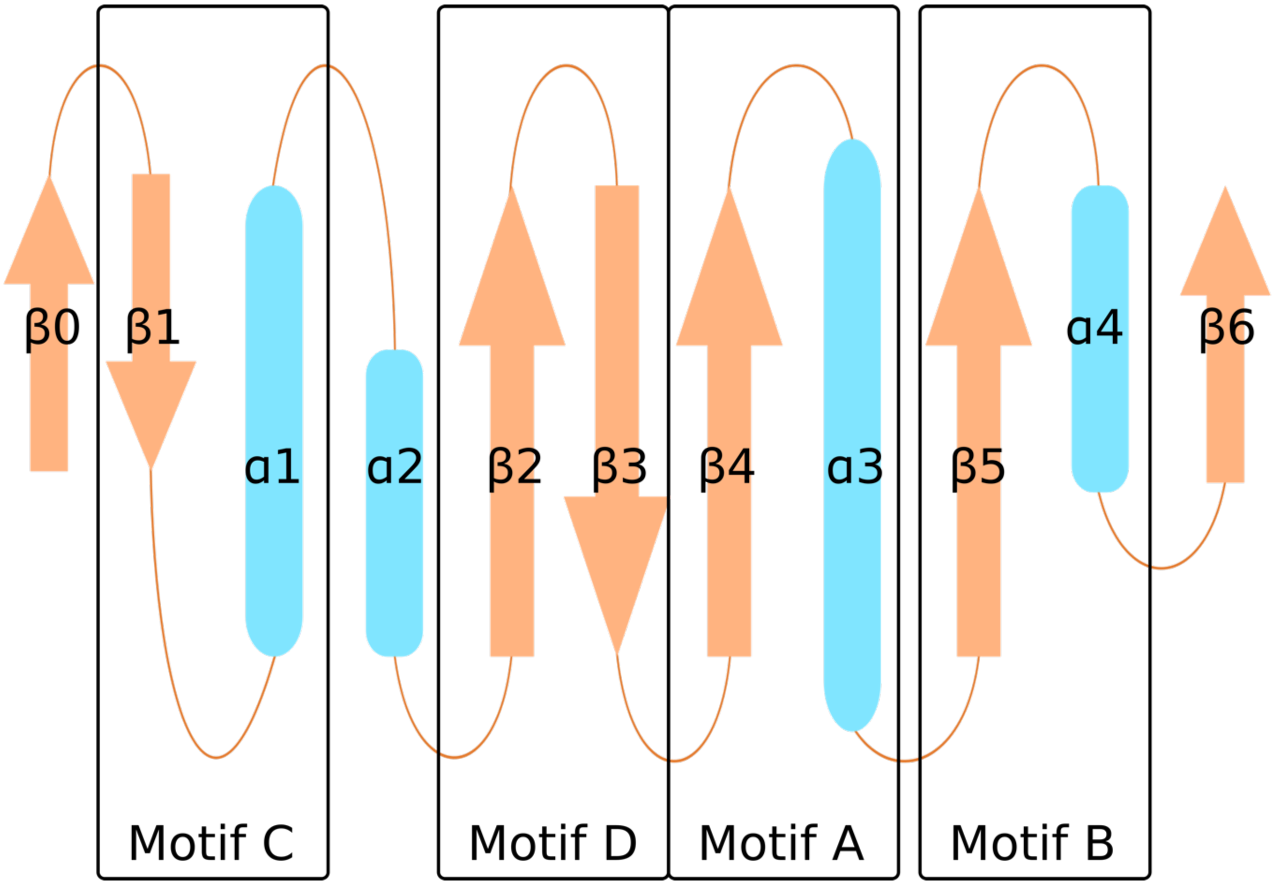

Figure A. Two out of four structural motifs on the GNAT fold are well conserved and suitable for phylogenetic analyses. Motifs A and B are well conserved over the entire acetyltransferase superfamily and are suitable to use for phylogenetic study. Unlike A and B, motifs C and D are not well conserved and not suitable for making MSAs between distant acetyltransferase sequences.

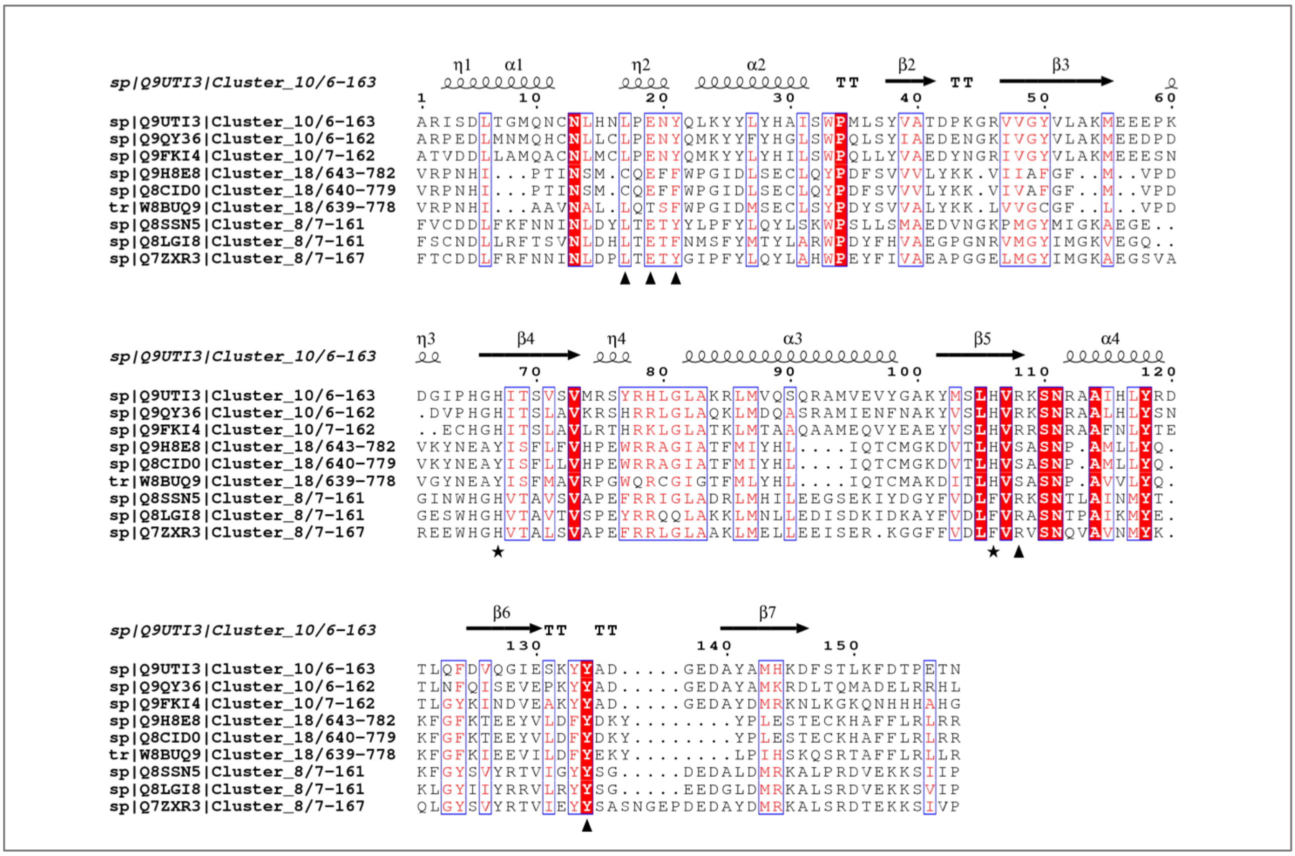

Fig B. MSA between NAA10 (cluster 10), NAA20 (cluster 8) and KAT14 (cluster 18). These sequences share a common ancestor, according to our phylogenetic tree. As such, they share a number of conserved residues. Residues marked with black triangles are important for substrate binding and catalysis in NAA10 and NAA20. In α1-α2 loop, glutamate plays a catalytic role in NAA10 and it seems to be conserved in KAT14 as well. Together with the catalytic role, it is involved in substrate binding. Another important residue in NAA10 and NAA20 is tyrosine in β6-β7 loop (Y134 in the alignment), which is involved in substrate binding and catalysis. This tyrosine is highly conserved in all Group 1 and Group 2 NATs, but also in KAT14. A black star under a position in the alignment marks a place where KAT14 has conserved residues equivalent to catalytic residues of NAA50 (tyrosine in β4 strand and histidine in β5) which are conserved in NAA10 as well but do not retain catalytic activity.

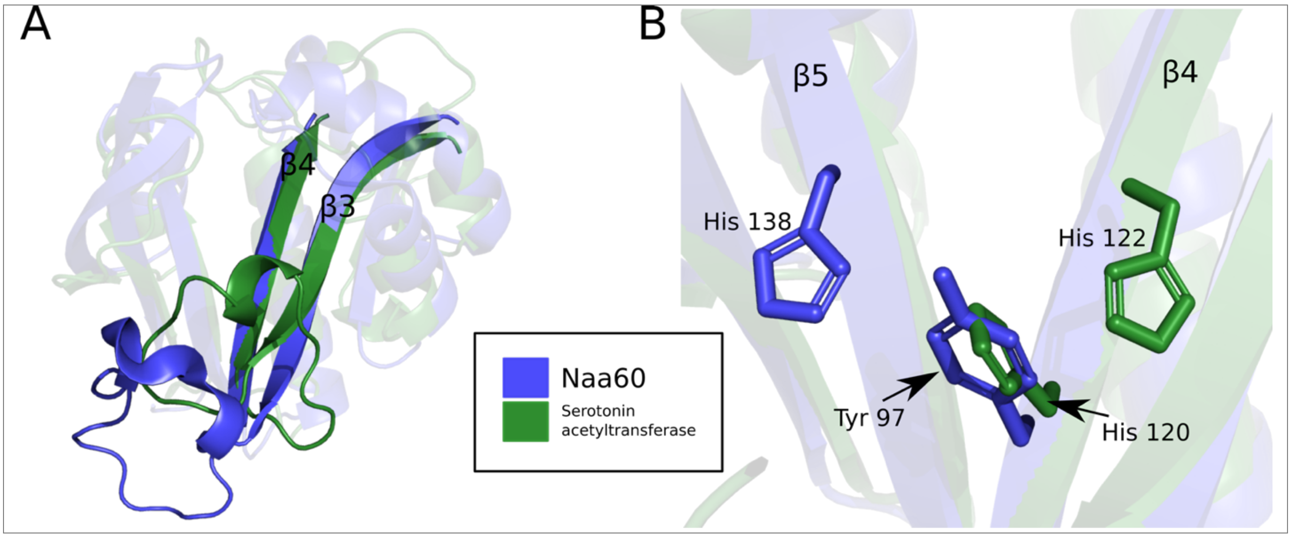

Fig C. Structural similarities between NAA60 and serotonin acetyltransferase. The typical β3-β4 loop of NAA60 exists in the serotonin acetyltransferase, as well (**A**). This loop is shorter in all other NATs. The position of catalytic residues is also similar. The position of Tyr 97 of NAA60 and His120 of serotonin acetyltransferase is the same (**B**). They are both found on β4 strand. The second catalytic residue of serotonin acetyltransferase is located on β4 strand, while the second catalytic residue of NAA60 is located on the β5 strand. Both of these residues are located on the characteristic V-shaped splay of the GNAT fold and close to the first catalytic residue.

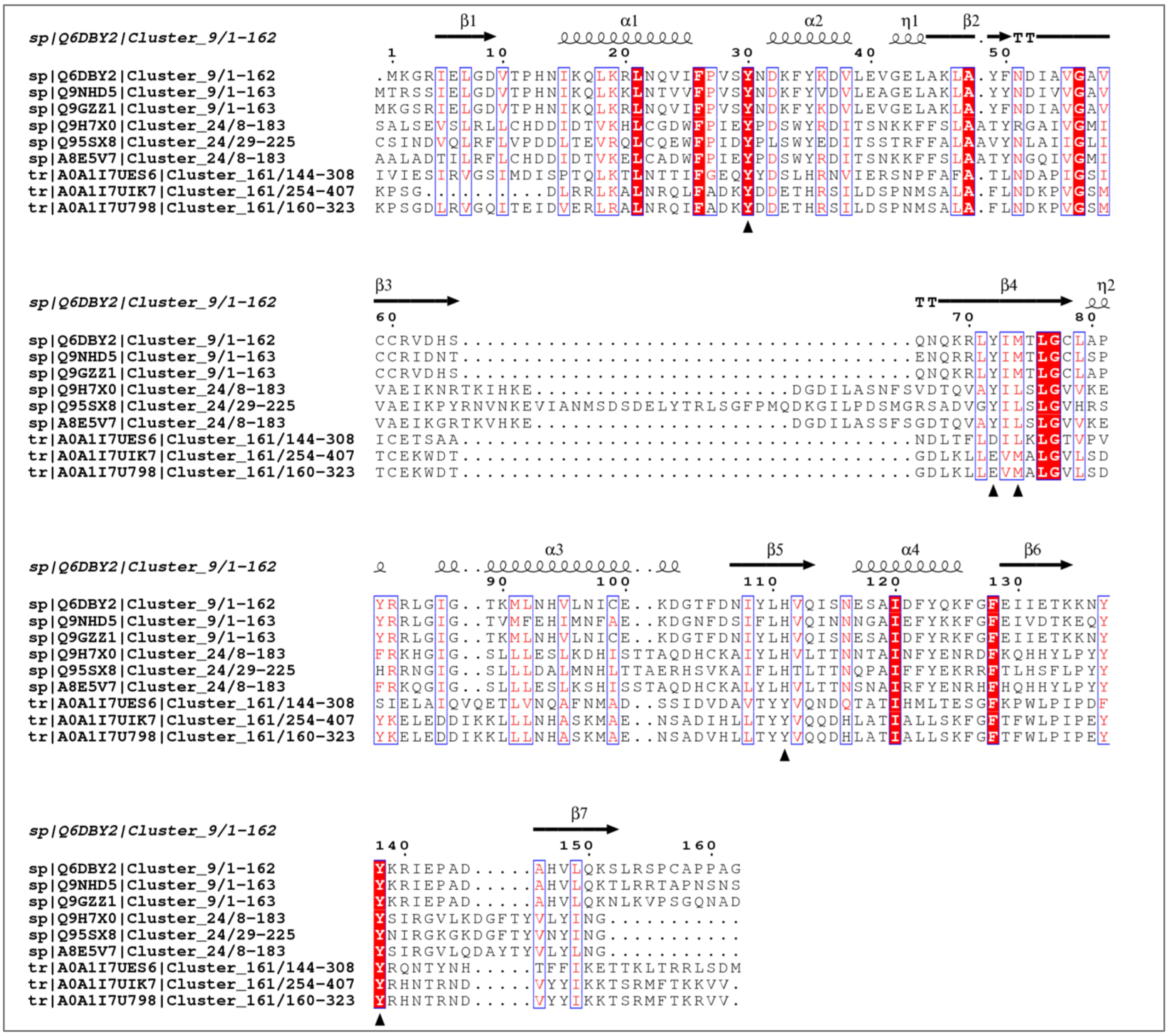

Fig D. Cluster 161 aligned to NAA50 and NAA60. A lot of key residues are conserved between NAA50, NAA60 and cluster 161. In α1-α2 loop, a tyrosine is conserved (Y30 in the alignment). It is involved in substrate binding in NAA50 and NAA60. A tyrosine in β4 in NAA50 (Y72 in the alignment) is a catalsytic residue. In cluster 161, in the same position there are negative charged residues which could act as catalytic residues as well. A tyrosine in the β6-β7 loop (Y138 in the alignment) is well conserved across all three clusters.

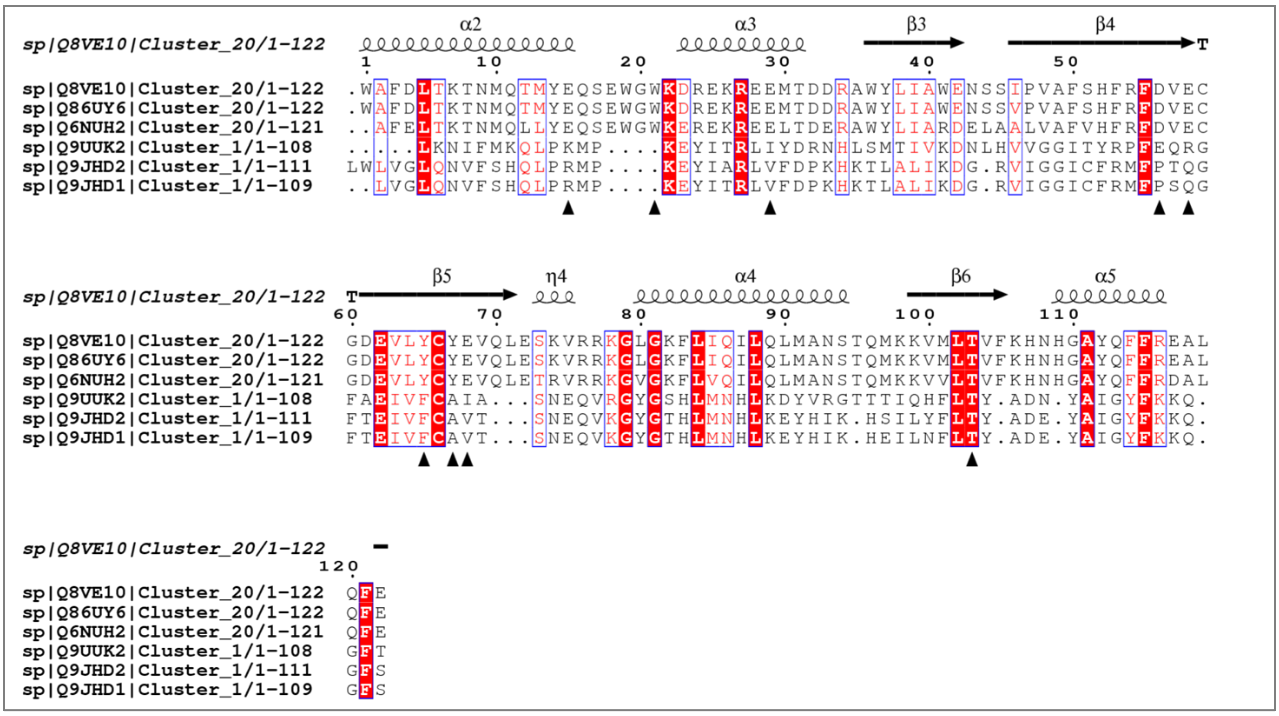

Fig E. MSA between NAA40 and cluster 1 histone acetyltransferases. There is high conservation on several sights in the alignment, but key substrate-binding and catalytic residues of NAA40 (marked with a black triangle) are not conserved in cluster 1 sequences.
