# Supplementary material for: Classification and phylogeny for the annotation of novel eukaryotic GNAT acetyltransferases
Source: PLoS Comput Biol. 2020 Dec 23;16(12):e1007988. doi: 10.1371/journal.pcbi.1007988 (PMC7790372; doi:10.1371/journal.pcbi.1007988)
Supplement: S6 Text — (PDF) [file pcbi.1007988.s006.pdf]

Network analyses: predictions of new NATs

Group 2 – NAA50 and NAA60

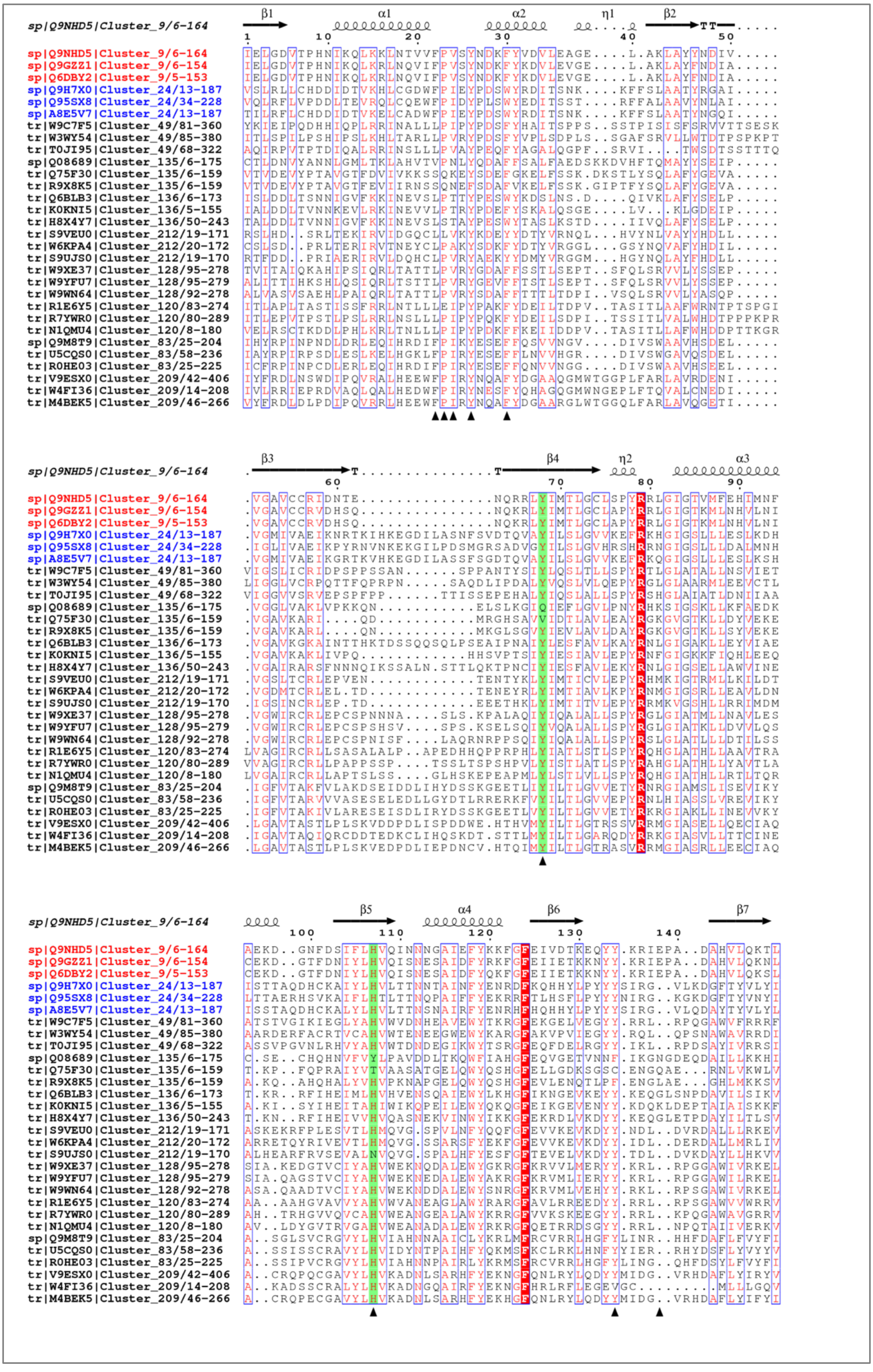

Fig A. Multiple sequence alignment of predicted NATs in Group 2. Alignment of NAA50 (cluster 9), NAA60 (cluster 24) and uncharacterized clusters surrounding these two NATs in the SSN. Residues marked with a black triangle are involved in substrate binding in NAA50 and residues highlighted green are involved in catalysis in NAA50. Catalytic residues are well conserved over the entire alignment, but there is a lot of variation in substrate binding residues from one cluster to another.

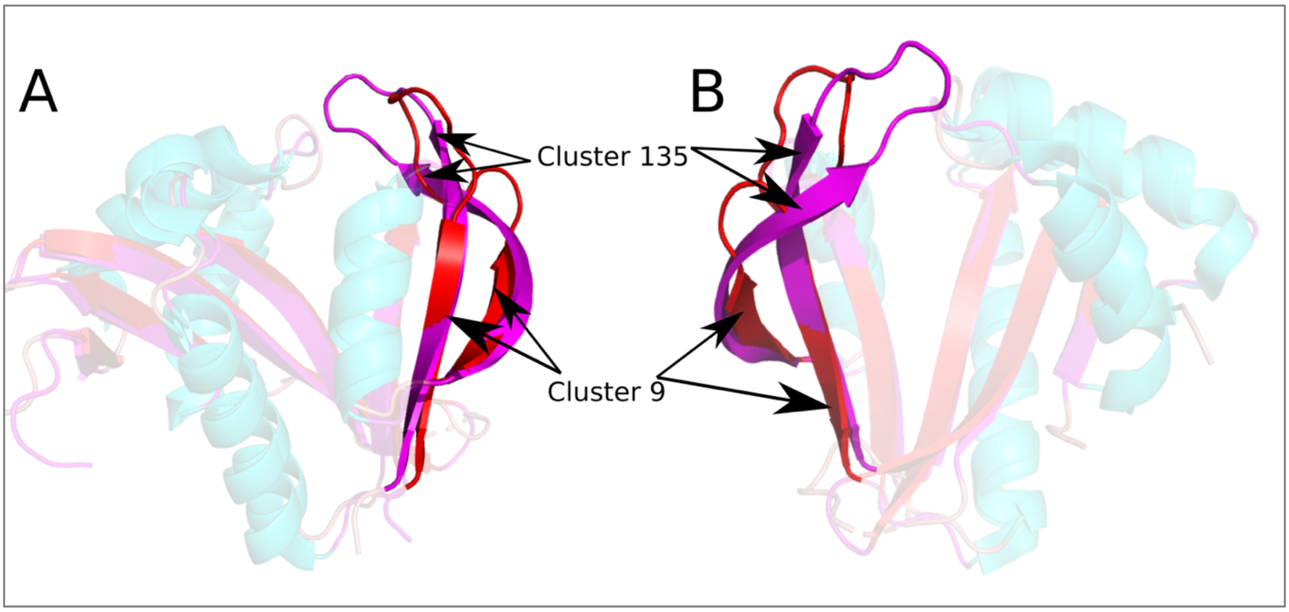

Fig B. Difference between NAA50 structure in clusters 9 and 135. The important β6-β7 loop differs between cluster 9 NAA50 and cluster 135 NAA50, which could be one of the reasons for their different specificities. Here, we show differences in size of this loop and of β6 and β7 strands on **A)** view from the back and **B)** from the front side of the GNAT fold. Cluster 135 strands and corresponding loop are colored magenta. Corresponding elements of cluster 9 are colored red.

7.2. Group 3 – NAA40

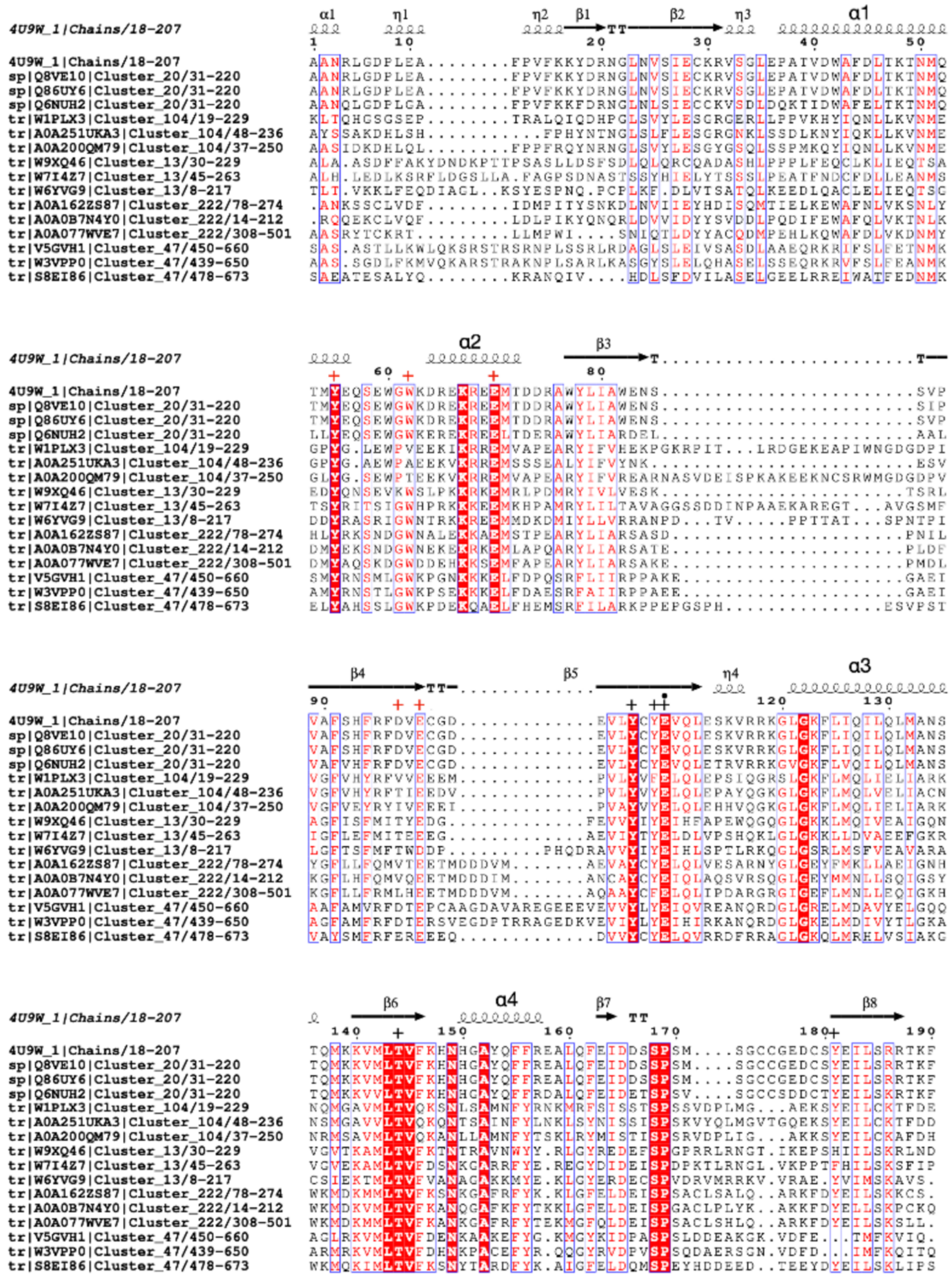

Fig C. Multiple sequence alignment of representative sequences from clusters 13, 20, 47, 104 and 222. Black plus sign (+) represents residues involved in substrate binding whose mutation doesn't affect catalysis in human NatD. Red plus sign (+) above residues means these residues are involved in substrate binding and their mutation significantly reduces acetylation levels (according to experimental results of Magin et al).

7.3. Group 4 – NAA80

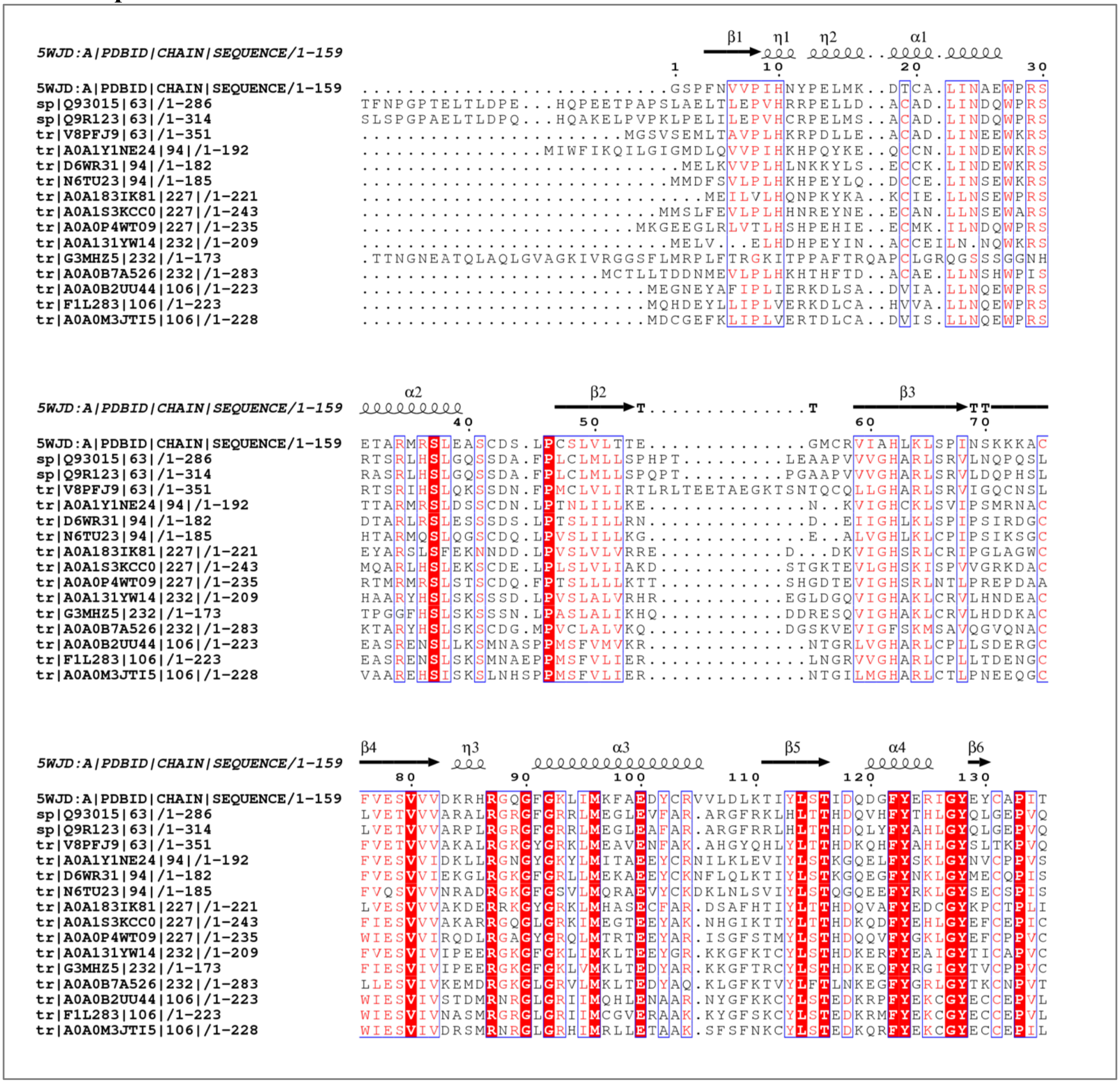

Fig D. MSA of NAA80 (cluster 63) and surrounding cluster found in SSN. The alignment shows high conservation across all sequences. It is most likely that clustering in this region was a result of taxonomical differences in sequences (most likely in N and C termini).
