# Supplementary material for: Classification and phylogeny for the annotation of novel eukaryotic GNAT acetyltransferases
Source: PLoS Comput Biol. 2020 Dec 23;16(12):e1007988. doi: 10.1371/journal.pcbi.1007988 (PMC7790372; doi:10.1371/journal.pcbi.1007988)
Supplement: S7 Text — (PDF) [file pcbi.1007988.s007.pdf]

Experimental verification of clusters 49 and 120

Cloning, expression and purification

The genes N1Q410 (Gene ID: DOTSEDRAFT_40460) and A0A194XTA9 (Gene ID: LY89DRAFT_680136) were synthesized by GeneArt (ThermoFisher Scientific, Germany). Both genes were subcloned into a pETM-11 vector (EMBL, Germany) using the restriction enzymes NcoI and Acc65I (New England Biolabs, USA) resulting in pETM-11-N1Q410 and pETM-11-A0A194XTA9, each expressing an N-terminal histidine tag for affinity purification. The plasmids were transformed into E. coli BL21 Star^{TM} (DE3) cells (Invitrogen) by heat shock. A 1 L BL21 bacterial culture was cultivated in Luria-Bertani (LB) medium at 37°C until an OD600 of 0.6 was reached and subsequently transferred to 20°C. Protein expression was induced by addition of 1 mM isopropyl-β-D-thiogalactopyranoside (IPTG). After 16 h, the cells were harvested by centrifugation and the pellets were stored at -20°C until further use. For purification, the E. coli pellets were thawed at 4°C and the bacterial cells lysed using mechanical disruption by a French Press (1000 psi pressure) in lysis buffer (20 mM HEPES (pH 8.0), 300 mM NaCl, 20 mM imidazole, 1 mM DTT, 1 mM PMSF, 1 x EDTA-free protease inhibitor cocktail (Roche)). After centrifugation (40,000 x g, 30 min, 4°C), the cell extract was applied on a metal affinity FPLC column (HisTrap HP, GE Healthcare). The proteins were eluted in a gradient with 300 mM imidazole in 20 mM HEPES (pH 8.0), 300 mM NaCl, 1 mM DTT, 1 mM PMSF. Fractions containing the recombinantly expressed protein were pooled and further purified via size exclusion chromatography (Superdex 75, GE Healthcare) in following buffer: 20 mM HEPES (pH 8.0), 300 mM NaCl, 1 mM DTT, 1 mM PMSF. The protein purities were determined by analysis of Coomassie stained SDS-PAGE gels. The protein concentrations were determined by absorption at 280 nm using a NanoDrop2000 spectrophotometer (Peqlab, Germany).

DTNB based N-terminal (Nt-)acetylation assay

The DTNB Nt-acetylation assay was performed as described in (Foyn et al. 2017). Briefly, purified enzyme (3 µM) was mixed with peptides (300 µM) and Ac-CoA (300 µM) in acetylation buffer (50 mM Tris-HCl pH 8.5, 200 mM NaCl, and 2 mM EDTA) at 37°C, and reactions were quenched after 60 min with quenching buffer (3.2 M guanidinium-HCl, 100 mM sodium phosphate dibasic pH 6.8). To measure CoA production, DTNB (2 mM final, dissolved in 100 mM sodium phosphate dibasic pH 6.8 and 10 mM EDTA) was added to the quenched reactions. The thiol present in the enzymatic product, CoA, cleaves 5,5′-dithiobis-(2-nitrobenzoic acid (DTNB) and produces 2-nitro-5-thiobenzoate (TNB^{1-}), which is readily quantified by monitoring the absorbance at 412 nm. Background absorbance was determined in negative controls (enzyme added after quenching buffer) and subtracted from the absorbance determined in each individual reaction. Thiophenolate production was quantified assuming $\varepsilon = 13.7 \times 103 \text{ x } M^{-1} \text{ x } cm^{-1}$.

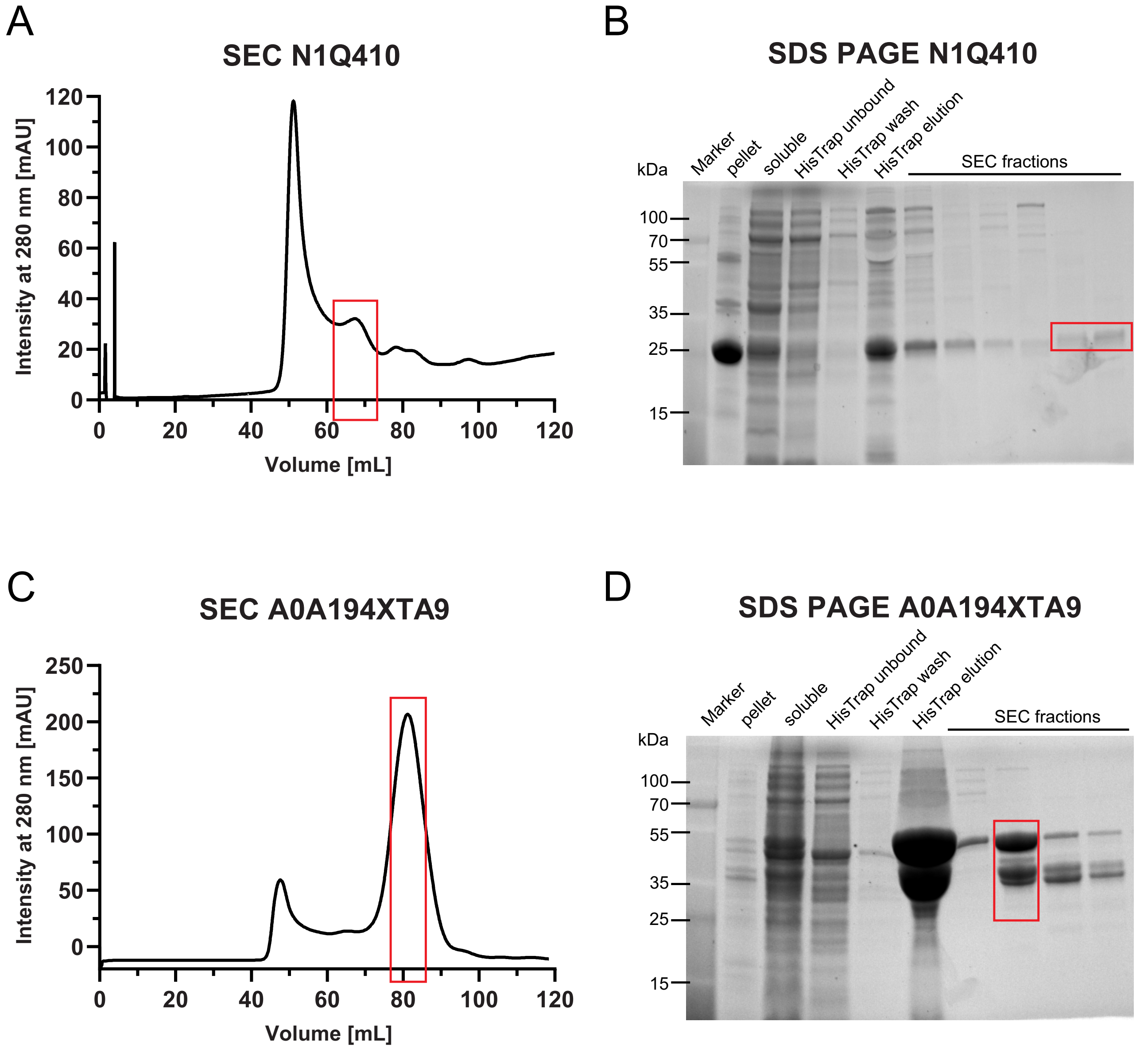

Fig A. Purification and DTNB-based activity assays of the putative NATs N1Q410 and A0A194XTA9. (A and C) Size exclusion chromatogram (SEC) and **(B and D)** coomassie-stained SDS PAGE gels from the purifications of N1Q410 **(A, B)** and A0A194XTA9 **(C, D)**. Highlighted in red boxes are the fractions that have been pooled, upconcentrated and further used for activity assays.
