# Supplementary figures and images for: Classification and phylogeny for the annotation of novel eukaryotic GNAT acetyltransferases
Source: PLoS Comput Biol. 2020 Dec 23;16(12):e1007988. doi: 10.1371/journal.pcbi.1007988 (PMC7790372; doi:10.1371/journal.pcbi.1007988)

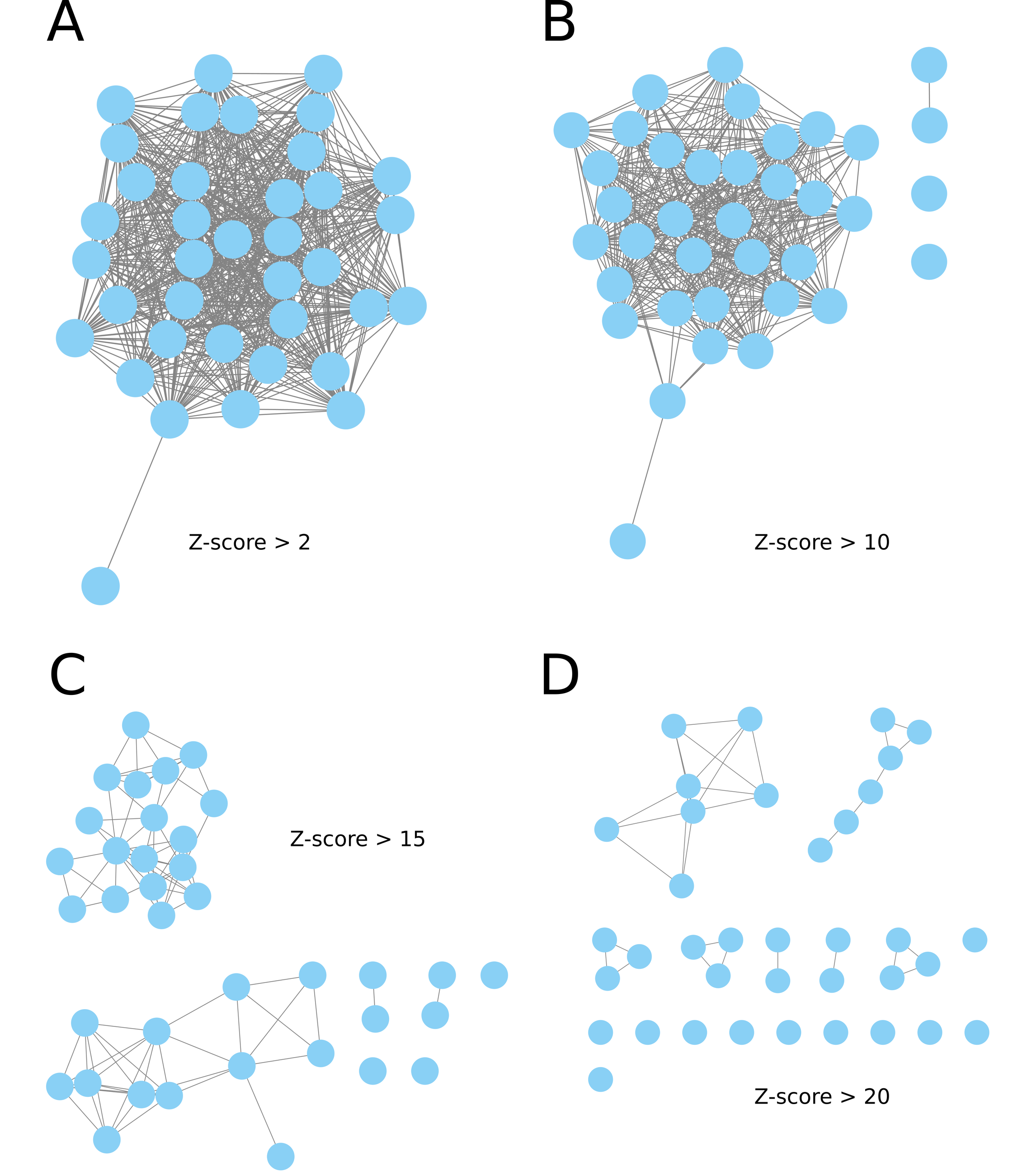

Supplement: S1 Fig — Acetyltransferases are structurally highly similar, which is reflected in the similarity networks and how they change as a function of the threshold Z-score. Networks constructed with Z-score > 2 and Z-score > 10 are random. First separation between nodes into different clusters is at Z-score > 15. (TIF) [file pcbi.1007988.s008.tif]
