# Supplementary material for: Classification and phylogeny for the annotation of novel eukaryotic GNAT acetyltransferases
Source: PLoS Comput Biol. 2020 Dec 23;16(12):e1007988. doi: 10.1371/journal.pcbi.1007988 (PMC7790372; doi:10.1371/journal.pcbi.1007988)
Supplement: Attachment — Submitted filename: KrtenicB_Response_to_Reviewers_R1.pdf [file pcbi.1007988.s019.pdf]

Reviewer 1

Krtenic et al's manuscript describes how they have classified the eukaryotic protein N-terminal acetyltransferases (NATs) into five groups using sequence similarity networks and characterized their sequences in terms of motif presence and phylogeny. I'm sure their classification is useful in the field, but a wider scope, primarily by including evolutionarily related sequences from bacteria and archaea would have made their contribution much more significant.

We thank the reviewer for their useful comments on our work, and the many suggestions that have helped us improve this manuscript and resolve potentially misleading statements. In what follows we also try to clarify why we think the data we present actually support our claims. In particular we would like to stress that the classification is not based on the phylogeny but on the SSN topology (see also answer to comment 2(b).

(1) Do the data and analyses fully support the claims? If not, what other evidence is required?
Not fully. The major shortcoming with the manuscript is the limitation to eukaryotic sequences. Besides reducing the generality of the findings, all genes in eukaryotes do not have the same evolutionary history -- some were likely vertically transferred from the last eukaryotic ancestor (and is hence likely shared with some modern Archaea), others have been introduced horizontally via the mitochondrion or chloroplast or other HGT events. It is possible that including bacterial and archaeal sequences would reveal further events like this besides the group 5 that the authors note is of bacterial origin. Without this, many of the claims in the manuscript are not very conclusive. (I note that the authors make the same suggestion, to include bacterial and archaeal sequences, in lines 602-4.)

Yet, and because the suggestion from Reviewer 1 is interesting, we have evaluated how the inclusion of bacterial and archaeal sequences would impact our claims and have come to the conclusion that it would not change the classification we propose for eukaryotic NATs (Cf Figure 1 below), and as a consequence it would not change the relationship between NATs revealed by the phylogenetic tree of the eukaryotic proteins. In what follows, we explain the rationale for this conclusion. At this point, we would also like to stress that our manuscript is not discussing evolutionary events and that the tree is only used for classification purposes.

The addition of archaeal sequences to our dataset for building the SSN would yield a new node linked only to node 10 (Naa10) in the simplified view (Cf Figure R1 below, and Figure 2 in manuscript). The sequences would belong to Group 1 as NAA10, NAA20 and NAA30, and the fingerprint would be similar to the one obtained without archaeal sequences since the amino

acid positions are strongly conserved between those enzymes. Adding archaeal sequences would not add new information to the proposed classification either.

Regarding the bacterial NATs, the addition of reviewed sequences to the network will not bring additional information because they will cluster separately from the eukaryotic ones. Four bacterial NATs have been confirmed thus far: RimI, RimJ, RimL and one non-Rim NAT. A BLAST search of RimI against SwissProt only yields 12 hits with low E-value. These include NAA50, NAA20 and NAA30 with ~30% identity but with only ~30% of query cover. The same applies to other Rim proteins; RimL retrieves only 17 hits against the nr database for example. SSNs are built from pairwise BLAST comparisons. Therefore, adding prokaryotic sequences to the SSN using the parameters combination we optimized for this superfamily will yield prokaryotic clusters isolated from the eukaryotic ones. We show the resulting SSN on **Figure R1** below. Adding reviewed bacterial sequences would thus not change the classification. Including all available (reviewed and unreviewed) prokaryotic sequences would results in a dataset of about 900000 sequences, with a high diversity and thus would not result in a more reliable phylogeny than what we present for the eukaryotes. Furthermore the tractability of an analysis of a dataset of that size is at best challenging.

For the purpose of annotating unknown bacterial sequences, if that should be interesting, one could simply blast a bacterial sequence of interest against our eukaryotic dataset and conclude on the protein function based on which cluster the hits belong to, if any.

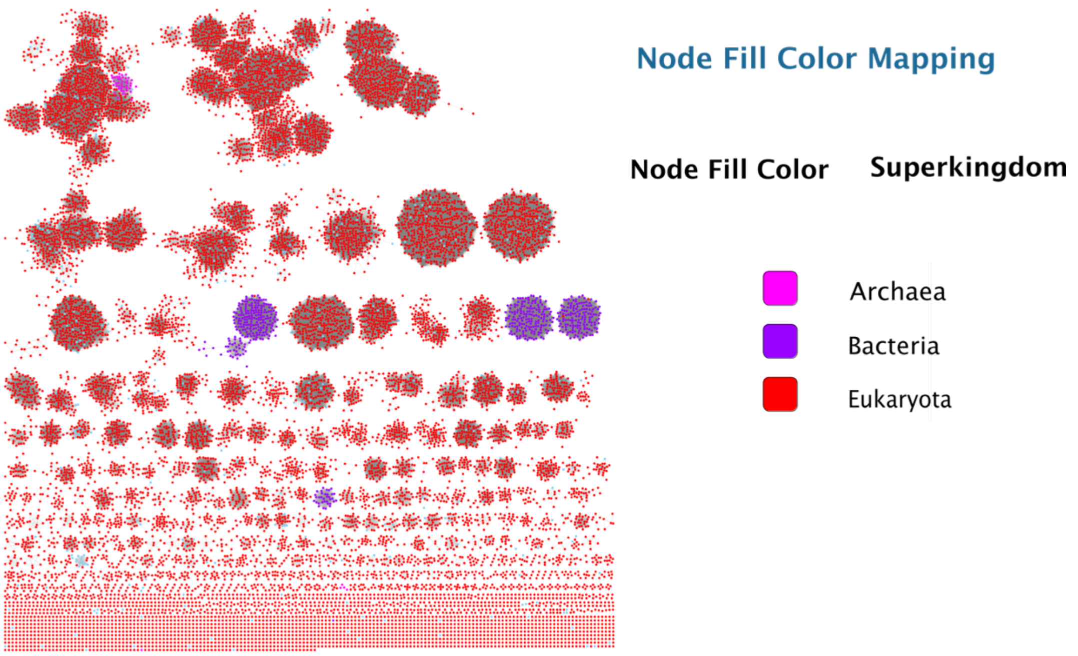

Figure R1. SSN calculated from the original dataset used in the manuscript augmented with the known prokaryotic NATs sequences, totaling ca. 15000 sequences. Purple clusters are RimI, RimJ, RimL and YiaC. They form single clusters distinct from the eukaryotic NATs. The magenta cluster is formed around the Archaeal NATs and establishes weak contacts with the eukaryotic NAA10, in agreement with what was reported earlier by Liszczak and Marmorstein (Liszczak and Marmorstein 2013).

(2) (a) Another shortcoming is the very limited information available in the alignment (75 amino acids,including several gappy positions) on which the authors build their phylogeny. There is possibly not much the authors can do about this, but if this is actually the case, perhaps a trustworthy phylogeny is not possible or the authors could try to show how robust their phylogeny is by adding some more positions to the alignment even though they might not be conclusively aligned (here I would also recommend using a tool to select trustworthy positions, e.g. BMGE (Criscuolo and Gribaldo 2010)) plus looking at possible conflicts in the data by e.g. phylogenetic networks.

We would like to reassure the reviewer that the phylogeny is based on the positions that lead the most reliable analysis of our dataset and that we have early on probed the robustness of the tree by varying the number of positions included. The GNAT fold, common to all proteins in our dataset, is 130-150 residues long on average and we restricted our alignment to the part of the fold that is conserved enough to give informative alignments. This is the result of trimming the alignments (as BMGE would do) but based on literature (Dyda et al. 2000; Vetting et al. 2005).

In addition the phylogenetic tree agrees well with the knowledge about acetyltransferase specificity available in the literature in particular to the work of Rathore et al (Rathore et al. 2016); known NATs do branch as expected given their substrate specificity. It also lead us to observe the proximity of acetyltransferases close to known NATs for which we didn't find evidence of similarity using other methods. An example is KAT14, a histone acetyltransferase which, as it turns out, shares one key sequence motif with Group 1 NATs. Finally the relationships between NATs found in the tree fit with the results from structure comparison using DALI, ie without sequence comparison.

(2) (b) If it proves impossible to show that the tree is robust and hence is not trustworthy, it might be better to basethe classification on sequence similarity alone.

We feel the need to clarify a potential misunderstanding: The classification of NATs into five groups does not depend on the tree topology but is obtained, as the Reviewer suggests, from sequence similarity by combining the SSN topology, sequence motif analyses and evidence from the literature. The tree topology merely reflects the classification we already established. We have modified the text in the Results section to make this clearer. The tree is used to help predict potential novel NATs or non-NAT enzymes that could have NAT activity. It also confirms the assumption based on the SSN that the same function evolved several times on the GNAT fold.

The following sentences were added (page 15, lines 317-320):
"The constructed tree is unrooted and does not inform on the direction of evolution within the superfamily. It therefore represents a network of similarity of acetyltransferases which reflects

well the classification of NATs we established earlier using a combination of SSN, sequence motif analyses and evidence from the literature. "

(2) (c) Moreover, despite the tree not being rooted, the authors make a number of claims for the evolution of different groups of sequences which, strictly speaking, are not possible to do based on an unrooted tree.

The reviewer reminds us that the unrooted tree cannot be used to determine the direction of evolution and it was not our intention to do this. Yet we have used unfortunate wording in a few statements. On lines 378 and 379, we claimed that NAA40 and NAA80 evolved from or with histone acetyltransferases. We cannot claim what these enzymes have evolved from based on an unrooted tree and we have removed this statement. We also use the term "common ancestor", which is strictly speaking not a correct term for an unrooted tree, but only for those clades that would get split by placement of a root. We have thus removed the term common ancestor and replaced it by "closely related taxa". The relationships between taxa, which are the focus of our analysis, remain the same regardless of the tree being rooted or not.

(3) Furthermore, in the phylogeny, group 1 is clearly not monophyletic. Although the authors make no claim to discover evolutionarily related groups, I think that it would be good to, after noting that the group is not monophyletic, revisit the classification to see if there is evidence in the SSN and motif search to split the group into two. The description of this might be easier to follow in a more reduced result section where all evidence for potential groups is first presented, then followed by a proposal (but see above for more comments regarding the disposition of the material). which in turn is followed by a discussion section.

Strictly speaking, we cannot conclude that group 1 is made of two monophyletic groups, since our tree is unrooted. Hence we find it sensible to keep in the manuscript the classification solely based on the SSN, meaning Group 1 consists in NAA10, NAA20 and NAA30.

To make it clearer for the reader that Group 1 is *a priori* not monophyletic, we have now emphasized in the text that NAA30 is not located on the same branch as NAA10 and NAA20 and that we have classified all NATs based on their sequence similarity and evidence from the literature. This has been added on page 15 lines 325-327.

The following sentence was added:
"NAA10 and NAA20 do not share the same branch with NAA30 but based on the sequence motifs on the key elements these tree NATs still belong to the same group."

(4) * Would additional work improve the paper? How much better would the paper be if this work were performed and how difficult would it be to do this work?

Potentially yes. Extend data with bacterial and archaeal sequences. This is of course a major undertaking, but would make results interesting for a larger group of readers as well as potentially reveal interesting evolutionary histories of some of the proteins. If the above mentioned problems with the phylogeny and alignment can be handled in a convincing way, adding sequences would potentially also allow the eukaryotic part of the tree to be rooted and stronger claims could be supported.

While we agree that being able to cover all domains of Life would tremendously increase the impact of this study, we need to stress that it is a strategy that will not lead to any reliable results given the nature of the dataset. This is due to the high dissimilarity between eukaryotic and prokaryotic sequences, and because well-studied acetyltransferases (serving as reference nodes in the SSN) constitute only an extremely small share of the dataset. These are some of the reasons why we have restricted our scope to eukaryotic NATs which are, in our opinion, the largest and most meaningful dataset to further our understanding of N-terminal acetylation in eukaryotes. Please also see ours answers to points (1) and (2) above.

(5) * Is the manuscript well organized and written clearly enough to be accessible to non-specialists?
Partly. The results section is written like a combined results and discussion section, then follows a second discussion section. I can see that this kind of manuscript is quite well suited for a combined results and discussion section, although the presentation of the phylogeny, which throws part of the classification in question (group 1 is not monophyletic), calls for a revisit of the grouping which might be more amenable to a shorter result section followed by a discussion section.

We appreciate that suggestion which is probably rooted in our description of the Results, which at times is extensive but justified by the amount of results. We are worried that a shorter Results section will result in moving more technical aspects to the Discussion section, which would in turn become inaccessible to the NATs scientist who are not all computational biologists.

(6) L29, 51 & 125: "homology" should be replaced with "sequence similarity".
This has been corrected.

(7) L35: "fungi" should be "fungal".
Corrected

(8) L70 & 168: "higher" and "lower" are unfortunate words in evolutionary discussions. What do you actually mean: multicellular eukaryotes or what?
We replaced higher and lower with multicellular and unicellular Eukaryotes on L70 and remove the expression on line 168 as it is not needed.

(9) L106: "The majority" not "Majority".

Corrected

(10) Figure 2: The colours of the groups in the legend to the right are not the ones used in the figure.

We checked the colours and the legend, and this figure was replaced. It was also modified following the comments from Reviewer 2.

(11) L206: "which extends over and covering the binding site" should be "extends over and covers"

Corrected

(12) L210: "has evolved to catalyzes N-terminal" should be "to catalyze"

Corrected

(13) L225: Should "length" be "height"?

Corrected

(14) L304: The manuscript did not specify if KAT14 has the Tyrosine involved in substrate binding (located in β6-β7 loop and present in group 1 and group2 NATs). It is then not clear if KAT14 might accommodate the same type of substrate or just a large dimension substrate (thanks to the presence of the β6-β7 loop).

We added the sentence: "The same tyrosine is present in the KAT14 β6-β7 loop (**Fig s17**)." on page 16 lines 340 and 341 to make it clear that the same tyrosine is conserved in KAT14.

(15) L517: Since the phylogenetic tree is based on a MSA that excluded the ligand specificity motifs (such as α1-α2 or β6-β7 loop), except for groups 3 and 4 (β4 and β5), it is not accurate to affirm that : "The phylogenetic tree (...) provides a useful perspective on the evolution of ligand specificities".

The expression "evolution of ligand specificities" has been replaced with the expression "differences between ligand specificities" on line 579.

(16) L581: "where the highly conserved F and V in the of NAA50 motif are replaced" : missing word? Or just extra "of"?

We have deleted "of"

(17) L633: "minimal sequence identity equal to of 40% on average" should be "equal to 40%"

Deleted "on average".

(18) L659: It is useful to mention if the network used here is weighted or unweighted (and what defines the edge weight), as the calculation of betweenness centrality is different.

This is a very good point. The network is not weighted, and it is undirected. This information has been added page 32 lines 731 and 732:

"Betweenness centrality was calculated on the representative, "pivot" network which is not weighted and not directed. "

Reviewer 2

This paper performed a superfamily wide bioinformatic characterization of the GCN5-related N-acetyltransferase (GNAT) superfamily. The bioinformatic pipeline that they used in this study sounds and provide a useful picture of the GNAT superfamily. I believe that the manuscript is suitable for publishing PLoS computational biology. I have only several suggestions to revise the manuscript to reach to wider communities.

We thank the reviewer for his careful reading of our manuscript and their useful comments.

(1) It is nice if the authors labeled other nodes with known enzyme function (in Figure 2, Figure 5 and related supplementary figures). I understood the focus of the work is studying NAT groups within the superfamily. However, mapping other functions would make this paper far more impactful and useful as it reaches to researchers that are working on non-NAT acetyltransferase in the superfamily. There are some in Figure 5, but it seems that there are more (as far as I can see in Figure 4). It would be nice if the authors showed all characterised clusters (regardless NAT or other substrates), and unexplored clusters. It is unclear the relationships between Figure 2 and 5. The should label cluster names as much as possible in Figure 5 to make it clear (in particular black lines). Similarly, it is informative if the authors label each PDB structure for associated cluster in the SSNs (Figure 2).

We thank the reviewer for this suggestion. We have labelled Figures 2, 4 and 5 as they suggested so that those three figures are more informative.

(2) Structural comparison - it is unclear how the authors compared the structure and what is the meaning of phylogeny (Figure 4). There is no description is available in Methods (just mentioned that used Dali Server). The authors should provide more detailed description in the text and Method, and explain the implication of the structural phylogeny, what is based on RMSD? what is the meanings of nodes and branches?

We apologize for this, we should have explained that Dali compares structures in a pairwise manner and using intramolecular distances calculated for each structure. No sequence information is being used in this comparison. The dendogram represents the degree of

similarity between the structures. We have now added that information in the Results section. (line 278-280 & 281-282) and more detailed information in the Methods section on page 33, lines 752-758:
"We used the DALI server to perform an all-by-all structural comparison of 38 unique structures of Eukaryotic acetyltransferase catalytic subunits. The comparison is done between selected intramolecular distances and no sequence information is used. The subunits are ranked based on the calculated pairwise similarities (Dali Z-scores) and a dendrogram that depicts the ranking is drawn (Holm et al. 2008; Holm and Laakso 2016; Holm 2019). The resulting dendrogram is simply a list of structural neighbors ranked by Dali Z-score. The list of PDB codes used for structural comparison is available in the supplementary material. "

(3) Again, I understood that the focus of the work is NAT, it would be nice to have a small discussion about how much clusters are uncharacterised and what would be substrates for those clusters.

We added information about the number of uncharacterized clusters in the Results section, page 8, lines 185-186:
"We identified 48 clusters with known acetyltransferases and 184 completely uncharacterized clusters."

Moreover we feel that we cannot conclude much else on the substrates for enzymes in these clusters. Because the GNAT fold seems to easily evolve new specificities one would need to thoroughly analyse the sequences of a cluster of interest to be able to conclude. We attract the attention of the reviewer to the following text in the Discussion section where we tried to convey this message L660 (in the revised text):

"Predicting the substrate specificity of an uncharacterized GNAT sequence which doesn't have close relatives with known function is practically impossible *in silico*. *In vitro* assays are necessary to map function and specificity of uncharacterized parts of the acetyltransferase superfamily. It is important to note that large portions of the phylogenetic tree have exclusively uncharacterized sequences and it is impossible to say anything about their substrate specificity."

Reviewer 3

The manuscript entitled "Classification and phylogeny for the annotation of novel eukaryotic GNAT acetyltransferases"describes a state-of-the-art computational approach for the functional annotation of the protein acetyltransferases that belong to the superfamily of GNAT acetyltransferases.
Their approach classifies the huge number of protein acetyltransferases into subgroups based upon specific structural features. This classification will be useful in future studies to predict and dissect the manifold acetyltransferases present in eukaryotes. The authors also provide a proof-of-concept for the predictive power of their classification system.

We thank the reviewer for their careful reading of our manuscript and their insightful comments.

(1) L73: ... has been shown that N-terminal acetylation affects ....
In this context, the very clear connection between N-terminal acetylation and diverse abiotic and biotic stress responses in plants should be mentioned
(Linster et al., 2015; Xu et al., 2015; Armbruster et al., 2020; Huber et al., 2020; Neubauer and Innes, 2020) reviewed in (Linster and Wirtz, 2018)

We thank the reviewer for noting this and have now added text in the introduction about the importance of Nt-acetylation in plants (Lines 76-79):
"The importance of N-terminal acetylation is also striking in plants, where it is involved in plant defense and development (Armbruster et al. 2020), response to abiotic stressors like osmotic and high-salt stress (Huber et al. 2020) and response to other various types of biotic and abiotic stressors (Linster et al. 2015; Xu et al. 2015; Linster and Wirtz 2018; Neubauer and Innes 2020)."

(2) L98 -: The NATs can be more or less promiscuous when it comes to substrate specificity (7).
This statement is not wrong but misleading to the non-expert reader. Please specify, "With respect to substrate specificity, some Nats are more promiscuous than others, e.g. NatB from fungi, plants and human share a narrow substrate specificity (MD, ME, MQ, MN) while other Nats possess more relaxed e.g. plant NatG or human NatF (Refs).
Here, the conservation of substrate specificities for distinct Nats should be introduced! Otherwise, the classification with respect to the catalytic subunits (Fig 2) makes not much sense.

We are grateful for this suggestion and agree that our sentence was misleading. We indeed needed more specific statements which are at lines 101-106 and 111-122.

"Some NATs are more promiscuous than others when it comes to substrate specificity (7). NatA is the most promiscuous NAT and it acetylates N-termini starting with A, S, T, C, V and G in fungi, plants and animals after the initial methionine is cleaved off (11,12,30). NatD and NatH, on the other hand, have only one type of substrate. NatD acetylates N-terminal serine (35,57), while NatH acetylates acidic N-terminus of processed actin (42,56). NatB, NatC, NatE, NatF and NatG have more relaxed specificity compared to NatD and NatH but are less promiscuous than NatA (58) "

"The conservation of specificity from fungi to animals is high for some NATs but is not fully established for all eight identified NATs. NatA and NatB, for example, have a quite well conserved specificity in all eukaryotes (11,12,30). NatE in has been shown to be catalytically inactive in yeast, unlike in multicellular eukaryotes where its specificity is well conserved (12,17,30,62). This has been shown only for yeast NAA50, however and it is unclear if all fungi NAA50s are catalytically inactive. NatC activity has been demonstrated in all eukaryotes (12,30) with specificity conservation between yeast and human (63,64) but plant NatC substrates still haven't been identified (65). While some NATs are present in all eukaryotic kingdoms, some NATs are not; NatF is not present in fungi, for example (38). NatG is located only in plastids of plant cells (41) and NatH has been identified only in animals (42)."

> *(3) The authors nicely compare features from animal and fungal Nats, but neglect the importance of the N–acetyltransferase machinery in plants. With the exception of NatG, the well characterized co-translationally acting Nats of the reference plants Arabidopsis thaliana are not mentioned throughout the result section.*

For most part, what is true for NATs of fungi and animals seems to be true for plant NATs as well. Our intention wasn't to compare NATs from different kingdoms but to give a general overview of characteristics for each distinct NAT and to compare those general characteristics. However, we agree with the reviewer that in some parts of the results we neglect useful information about the differences in plant NATs. We have now added more information in the relevant paragraphs of the Results and Discussion sections. We have also included the latest findings on 8 *A. thaliana* GNAT acetyltransferases with demonstrated N-terminal activity.

On pages 5 and 6, lines 111-113: "Besides Glucosamine 6-phosphate acetyltransferases, dual N-terminal activity has been demonstrated in plant plastids, where 8 GNAT acetyltransferases have been shown to be able to acetylate side chain lysines and protein N-termini (61)."

On page 10, line 219: "Group 1 contains NAA10, NAA20 and NAA30 of all eukaryotic kingdoms." This is to emphasize that when we write about these three NATs, we take all kingdoms into account.

On page 10 and 11, lines 229-233: "It is important to mention that NAA60 of animals and plants do not share the same cluster (**Fig s6**). Plant NAA60 bears some slight, but potentially important differences on the α1-α2 loop where a negatively charged E in animals is replaced by a positively charged K in plants. The key tyrosine of β6-β7 loop is still present in plants, but unlike animal NAA60 which has thee tyrosines in this loop, plant NAA60 only has one." This was added to emphasize the difference in clustering and potential difference in specificity of NAA60 between plants and animals.

The differences between plant and animal NAA60 have already been discussed on page 20, lines 435-442. We have made one slight adjustment to make it clear for the reader that the protein in question is from plants.

On page 11, lines 233 and 235 "Some plant NAA40 form a separate cluster and some others share the same cluster as animal NAA40 sequences."

On pages 17 and 18, lines 383-392: "Eight enzymes in *A. thaliana* mitochondria and chloroplasts have been shown to be able to acetylate protein N-termini (61). Three out of those eight enzymes are present in our phylogenetic tree (cyan branches) (**Fig 5**). Clusters 36, 71 and 82 are closely related according to our tree, which is in agreement with observations made by Bienvenut and colleagues in their study (61), where they group these three enzymes into the same subgroup. Interestingly, clusters 36, 71 and 82 are found branching close to Glucosamine -6-phosphate acetyltransferase (**Fig 5**), which has also been shown to be able to acetylate protein N-termini (60). The remaining five *A. thaliana* plastid enzymes with demonstrated N-terminal acetylation activity are found in clusters 50, 73, 74, 88 and 97 but they are not present in the tree since their inclusion lowers the robustness of the phylogenetic tree due to their high dissimilarity to the rest of the superfamily. "

On page 20, lines 444-446 "Cluster 83 contains only plant sequences while cluster 24 contains only animal sequences. The differences in sequence motifs we observe could indicate differences in NAA60 specificities between plant and animal NAA60."

On page 22, lines 472-477 "Cluster 20 contains the well characterized animal NAA40, but also sequences from the plant phylum Chlorophyta. Other plant sequences found in this group form cluster 104 and belong to the phylum Streptophyta. Fungal sequences form cluster 47 and 222 and do not mix with plant and animal sequences. The observed differences between cluster 20 and 104 indicate that fungal, plant and animal NAA40 might have different specificities."

On page 24, lines 518-522. "However, the recent study that demonstrates N-terminal acetylation activity of eight enzymes in *A. thaliana* plastids (61) might indicate the existence of more than 5 groups of NATs. We refrain from assigning more NAT groups based on this most recent discovery since the ability to acetylate N-termini doesn't necessarily mean N-terminal acetyltransferase function."

On page 27, lines 604 and 605: "…which has been indicated in a recent finding that *A. thaliana* serotonin acetyltransferase has weak N-terminal acetylation ability."

(4) L185: 26-NAA50 should be 24-NAA50 according to the Figure. Does this cluster also contain the inactive yeast NAA50?

This is an unfortunate typo which we now have corrected.
The information about the inactive NAA50 is now part of Figure 2 and clarified in the corresponding legend.
"Of importance is also cluster 135, which contains the catalytically inactive yeast NAA50."

(5) L 196: … "we defined five different groups of NATs (Fig 2)." This is misleading since Figure 2 shows only 4 groups.

It is indeed misleading. We have now modified the text to avoid any confusion (page 10, lines 215-217):
"…we defined four different groups of NATs (Fig 2.). We defined an additional fifth group based on the experimental evidence that NatG localizes in plastids of plants and has distinct substrate specificity."

(6) L 199: Group 1 contains NAA10, NAA20 and NAA30. NAA10 and NAA20 are in the same connected component, while NAA30 is found in a single isolated cluster." If NAA30 is part of the group 1, it should be shown in Fig2
We have added the node corresponding to NAA30 on Figure 2.

(7) L 203 "Group 2 consists of NAA50 and NAA60" Does the group also consist of catalytically inactive NAA50 from yeast? Please discuss.

This is a very good point indeed. Group 2 contains indeed the inactive yeast NAA50. This NAA50 is located in cluster 135 (see Figure 2 in the main text). We have now added the following in the Results section (page 10, Lines 225-229):
"Group 2 also contains a catalytically inactive yeast NAA50, a member of the fungal *Saccharomicetaceae* family (**Fig s11**). This inactive NAA50 forms a separate cluster, numbered 135, in the SSN (**Fig 2**). Sequence motifs on key secondary structure elements are not strictly conserved between cluster 135 and catalytically active NAA50 (cluster 9) explaining why the inactive enzyme clusters separately from cluster 9."

In addition, we have added a figure in the supplementary material (**Fig s11**)

Moreover we have added the following text to the Discussion section (Page 25, lines 532-541):
"Interestingly, Group 2 contains an inactive yeast NAA50 (found in cluster 135), which doesn't contain the characteristic β6-β7 tyrosine involved in substrate binding and present in Group 1 and Group 2 NATs (Deng et al. 2019). The inactive yeast NAA50 does not have the Ac-CoA binding motif either (Deng et al. 2019). It is therefore expected that its function is not

the same as NAA50 sequences which have all substrate interaction sites conserved. By comparing sequences **(Fig 6A)** and available structures **(Fig s22)** we found that these important binding sequence motifs are absent only in the *Saccharomycetaceae* from the fungal *Ascomycota* phylum. Other families from this phylum and members of other fungal phyla seem to have conserved substrate binding sequence motifs when compared to NAA50 of other eukaryotic kingdoms."

(8) L 233 "This tyrosine is essential for function and is strictly conserved in all members of groups 1 and 2 (43–45,48,68)." Please include information about the origin of catalytic subunits (fungi, plantae, animalia)

Thank you for pointing at this. We have added the following to that sentence (now lines 266-267): "with the exception of the catalytically inactive fungal Sacchormycetaceae NAA50 where this tyrosine is lost." to clarify that the tyrosine is found in all kingdoms and that the only exception is this family which contains an inactive NAA50.

(9) L 268: 4u9vA_NAA40-Q86UY6 is separated from group 1-4. This contradicts the main message of figure 4. Is there any explanation why it is so different from 4u9wA_NAA40_substrate? E.g., lower resolution of structure, or artificial crystallization conditions -> if so, please skip

We are extremely grateful that the reviewer noticed this, it prompted us to carefully go through the dendogram on Figure 4 again. We realized an error we made in our input structures (wrong chain identifier) for DALI when comparing the PDB files. We have now corrected this mistake and re-calculated the dendogram. We have double-checked other structures too and have updated the manuscript. Both NAA40 structures (4u9v and 4u9w) are now on the same branch.

Accordingly, we modified the text in the results section describing the dendogram and removed the following sentences from the last paragraph (Lines 293-297):
"One indication of how small the differences are between structures is the fact that the structures of NAA40 with and without substrate are found to be very distant from one another in the dendrogram (blue branches on **Fig 4**). This is the result of the position of the β6-β7 loop which is opened without the substrate and closed with the substrate bound to the enzyme (Magin et al. 2015)."

(10) L 298 Please also discuss the yeast NAA50 in this respect.

We have added "with the exception of the fungal Saccharomycetaceae family." to this sentence (Line 340 in the revised manuscript).
