# Supplementary material for: Classification and phylogeny for the annotation of novel eukaryotic GNAT acetyltransferases
Source: PLoS Comput Biol. 2020 Dec 23;16(12):e1007988. doi: 10.1371/journal.pcbi.1007988 (PMC7790372; doi:10.1371/journal.pcbi.1007988)
Supplement: Attachment — Submitted filename: KrtenicB_Response_to_Reviewers_R2.pdf [file pcbi.1007988.s020.pdf]

Reviewer 1

I confess I was confused when performing my first review of Krtenic et al.'s manuscript, and I still am, despite the efforts of the authors in this revised manuscript.

Let me summarise how I interpret the data presented to hopefully clarify any further misunderstandings. 1) Eukaryotic NAT sequences were clustered based on similarity. Some of the identified clusters contain members of known function (named NAAnn). 2) Each cluster was searched for sequence motifs which served as the basis for grouping some clusters by identification of similarities in motifs. 3) To investigate if the sequence similarity clustering is reflected in structural similarity, a distance-based phylogenetic tree was built from structural similarity scores. 4) A maximum likelihood phylogeny (not a "sequence similarity network" as the authors suggest to call it) was estimated from an alignment of structurally conserved regions of the sequences. Given the absence of an outgroup, the tree was not possible to root.

This is correct.
Note that the sequence motifs (2) are a novel result in themselves and will be useful for the NATs community of researchers.
We are thankful that Reviewer 1 thoroughly read our manuscript and our answer to their earlier comments. We also appreciate the detailed advices that help us improve this manuscript.

While the structural similarity tree is consistent with the classification in steps 1 and 2, the phylogeny does not support what the authors claim. This is not because the tree is unrooted, but because there is no possible way to root it and claim that "the branching in the tree clearly reflects the four different groups of NATs" (lines 321-2), "Group 1 and Group 2 are closely related according to the tree" (lines 323-4) and "these enzymes [group 1] are obviously closely related" (lines 528-9) (among other similar claims). The orange and purple parts of group 1 can't share a common ancestor that makes the two any more closely related to each other than to some other branches in the tree. What I see is that there is possible phylogenetic evidence for all the other groups – i.e. under reasonable rooting scenarios there's monophyly for the groups – but not for group 1. There are different possible evolutionary explanations for this: early divergence from an ancestor with the group 1 motif, combined with divergence in other descendants is one, convergence is another. (The suggested archaeal common ancestor to NAA10 and NAA50 would fit well, and the tree suggests inclusion of NAA20, NAA60 and, possibly, NAA30 as descendants of an archaeal gene.)
Let me be clear that I'm not against presenting a classification based solely on steps 1) and 2), followed by an analysis based on phylogeny that investigates possible scenarios for the evolution of the proposed classes and groups, but there are claims in the text that I do not find supported by the data. An alternative is, as I suggested in my first review, to revise group 1 based on the phylogeny into two: NAA10 and NAA20 on the one hand and NAA30 on the other. Since NAA30 is not

connected to any other cluster in the group in the SSN (fig. 2), separating it out does not seem to invalidate the principles behind the classification.

We originally grouped Naa10-Naa20 together with Naa30 in one group because of the similarity between their motifs. We understand the arguments of the reviewer have them divided in two groups, Naa10-Naa20 on one hand and Naa30 on the other hand. We have done so and modified the text and figures accordingly. To reflect the fact that Naa10-Naa20 and Naa30 show the same sequence motifs, we have called these two groups Group1a and Group1b. The text in all relevant sections of the manuscript was updated. In particular we modified the text related to the phylogeny to reflect the reviewer's comment that there is no evidence for a Group1a and Group1b to be closely related. Figures 2 to 5 were modified, and so was Table 1. We also updated the Supplementary Material to reflect this change.

I'm glad the authors performed a separate analysis to show what an analysis of bacterial and archaeal sequences might look like. Extending the analysis to all three domains is, if at all possible, clearly an overwhelming task.

We appreciate the fruitful discussion we are having with the reviewer.

I fully accept the authors choice to keep the structure of the manuscript as a combined results and discussion manuscript. However, I think it would be fairer to call the current results section "Results and discussion" as that makes it clear that what follows is not just the authors' own results but quite substantial discussion (evidenced also by the number of references). Given the length of the manuscript, ending with a Conclusions section would be helpful. This might mean moving some of what is in the current Discussion section to the R&D and some other material to the new Conclusions section. (For my taste, I think the M&M contains too much results, but I'm willing to turn a blind eye to this given the current length of the Results section.)

We now better understand the reviewer's suggestion and agree that restructuring would improve the readability of the manuscript. We have renamed the former "Results" section as "Results and Discussion", section to which we now have moved significant parts of the earlier Discussion section. The "Discussion" section from R1 is now shorter, polished and renamed "Conclusion" section.

The manuscript is very long. Besides reworking the manuscript as suggested above (much of what is in the current discussion could be worked into already started arguments in the current results section), shortening should be possible in places; subsection 5.1 strikes me as a good candidate.

As indicated by the reviewer restructuring the manuscript as mentioned above did result in shortening it somewhat. We also agree with the reviewer that Section 5.1 (4.1 in the revised version) is long and that readability could be improved. We have reformulated several sentences and reorganized paragraphs to increase clarity, and shortened the whole

section, but since it contains a fairly detailed analysis of the clusters neighbouring known NATs in the SSN, the change in size might not look drastic.

There are quite a number of typos and mistakes in the manuscript. I comment below on the ones I've found, but there are likely more of them.

- ***Line 175: "closely related" should probably be "very similar"*** : corrected
- ***Line 208: The cluster containing NAA30 is shown in the*** *figure* : corrected
- ***Line 210: "NATs" should be "NAT"*** : corrected

- ***Lines 258-9: You only specify what black and red mean.*** We have updated the legend of Figure 3.

- ***Lines 282-3: See discussion above.*** Corrected

- ***Figure 4: This tree is also inherently unrooted, but drawn as a rooted tree. Moreover, it's drawn as a cladogram so that branch lengths are not informative. In order to judge anything about closeness, you should at least change so that the tree is drawn with proper branch lengths, i.e. branch lengths that reflect the structural differences you used to generate the tree.***
  We respectfully disagree, there must be a misunderstanding. Figure 4 does not represent a tree (or a phylogeny) but a dendrogram (see figure legend). It results from the hierarchical clustering based on the matrix of Z-scores from DALI. Branch length is irrelevant. We have clarified this in the Methods section.

- ***Line 317: I think you should say here that the tree is not rooted because you don't have an outgroup.*** We changed this sentence to (now lines 367 and 368):
  "The constructed tree is not rooted because of a lack of a good outgroup and it does not inform on the direction of evolution within the superfamily."

- ***Lines 323, 326-7, 396: See discussion above.*** We removed the sentences about the proximity of groups 1 and 2 in the legend of Figure 5 and elsewhere.

- ***Figure 5: Please add the new, non-NAT, colours to the legend.*** The colors of the branches for glucosamine 6 phosphate and serotonin acetyltransferases, as well as the grey shading, have been added to the legend.

- ***Lines 398-9: Delete ", and for which we cannot infer functions based on our computational approach". (You do make attempts to form hypothesizes about sequences that are in clusters without any characterized enzymes.)*** : done
- ***Line 407: "NATs" should be "NAT"*** *:* corrected

- ***Line 510: I'm not convinced that "topology" is the best word here, it leads my thoughts to protein structure.*** This sentence was removed as a result of reorganizing the manuscript.

- ***Lines 579-80: What do you mean with "This observation indicates different evolutionary paths for NATs, and not divergent evolution"?*** We remove this sentence as it conveyed the same meaning as the next one (which is also more precise) namely

that "Our results indicate that N-terminal acetyltransferases evolved more than once on the GNAT fold".

- ***Line 694: Do you have a reference for saying that 70% "usually indicates shared function"? In which protein family? (I have certainly seen proteins with much lower degree of identity that have the same function.).*** This must be a misunderstanding. This sentence does not mean that proteins sharing less than 70% sequence identity do not share the same function. 70% is our upper threshold. We intended to convey the idea that 70% sequence identity is a safe choice to ensure shared function when reducing the size of the dataset. We have added a few references:
  Devos D, Valencia A. 2000. Practical limits of function prediction. Proteins Struct. Funct. Genet. 41:98–107.
  Rost B. 2002. Enzyme function less conserved than anticipated. J. Mol. Biol. 318:595–608.
  Tian W, Skolnick J. 2003. How well is enzyme function conserved as a function of pairwise sequence identity? J. Mol. Biol. 333:863–882.

- ***Line 700: "sequences clustering together were closely related": Perhaps I'm overly picky now, but you're talking about sequence similarity, not evolutionary relationships.*** We disagree here. We would not modify this statement as E-values and alignment scores for a hit in a database search are commonly used indicators of homology.

- ***Line 717: "as" should be "at"*** corrected
- ***Line 731: "using algorithm" should be "using the algorithm".*** corrected
- ***Line 749: "NAT" should be "NATs"*** corrected
- ***Lines 753 and 755: "is" should be "was".*** corrected

- ***Lines 756-7: Delete "The resulting dendrogram is simply a list of structural neighbors ranked by Dali Z-score." It's not a correct description of a distance-based phylogenetic tree (definitely not a "list").*** See comment above. This is a dendogram, not a distance-based phylogenetic tree. The legend of Figure 4 and the Methods section have been updated to clarify this but we are reluctant to write more as this is common knowledge in protein bioinformatics.

- ***Lines 775-6: "MAFFT" is incorrectly spelled in two places.*** corrected

- ***Lines 792-3: Delete "The tree was not rooted." Phylogenies that come out of programs are never rooted; you root them e.g. by including an outgroup.*** done

We have carefully read the manuscript and corrected all other typos we could find.
